# A tryptophan-derived uremic metabolite/Ahr/Pdk4 axis governs skeletal muscle mitochondrial energetics in chronic kidney disease

**DOI:** 10.1172/jci.insight.178372

**Published:** 2024-04-23

**Authors:** Trace Thome, Nicholas A. Vugman, Lauren E. Stone, Keon Wimberly, Salvatore T. Scali, Terence E. Ryan

**Affiliations:** 1 Department of Applied Physiology and Kinesiology and; 2 Division of Vascular Surgery and Endovascular Therapy, University of Florida, Gainesville, Florida, USA.; 3 Malcom Randall VA Medical Center, Gainesville, Florida, USA.; 4 Center for Exercise Science and; 5 Myology Institute, University of Florida, Gainesville, Florida, USA.

**Keywords:** Muscle biology, Nephrology, Chronic kidney disease, Mitochondria, Skeletal muscle

## Abstract

Chronic kidney disease (CKD) causes accumulation of uremic metabolites that negatively affect skeletal muscle. Tryptophan-derived uremic metabolites are agonists of the aryl hydrocarbon receptor (AHR), which has been shown to be activated in CKD. This study investigated the role of the AHR in skeletal muscle pathology of CKD. Compared with controls with normal kidney function, AHR-dependent gene expression (*CYP1A1* and *CYP1B1*) was significantly upregulated in skeletal muscle of patients with CKD, and the magnitude of AHR activation was inversely correlated with mitochondrial respiration. In mice with CKD, muscle mitochondrial oxidative phosphorylation (OXPHOS) was markedly impaired and strongly correlated with the serum level of tryptophan-derived uremic metabolites and AHR activation. Muscle-specific deletion of the AHR substantially improved mitochondrial OXPHOS in male mice with the greatest uremic toxicity (CKD + probenecid) and abolished the relationship between uremic metabolites and OXPHOS. The uremic metabolite/AHR/mitochondrial axis in skeletal muscle was verified using muscle-specific AHR knockdown in C57BL/6J mice harboring a high-affinity AHR allele, as well as ectopic viral expression of constitutively active mutant AHR in mice with normal renal function. Notably, OXPHOS changes in AHR^mKO^ mice were present only when mitochondria were fueled by carbohydrates. Further analyses revealed that AHR activation in mice led to significantly increased pyruvate dehydrogenase kinase 4 (Pdk4) expression and phosphorylation of pyruvate dehydrogenase enzyme. These findings establish a uremic metabolite/AHR/Pdk4 axis in skeletal muscle that governs mitochondrial deficits in carbohydrate oxidation during CKD.

## Abstract 2

In mice with chronic kidney disease, the degree of aryl hydrocarbon receptor activation and uremic metabolite accumulation drives muscle mitochondrial dysfunction via PDK4 upregulation, which decreases mitochondrial pyruvate oxidation.

## Graphical abstract

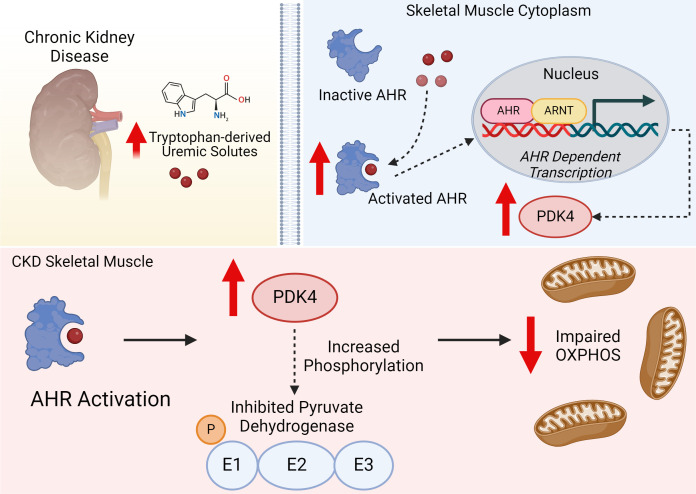

## Introduction

Chronic kidney disease (CKD) affects over 500 million people globally (1). CKD results in a progressive skeletal myopathy characterized by reduced muscle mass and strength, increased fatiguability, and exercise intolerance (2–5). The imbalance between muscle catabolic and anabolic pathways have been well documented in CKD; these pathways include the overactivation of the ubiquitin proteasome system, dysregulation of autophagy, increased caspase and calpains, and impaired insulin growth like factor 1 (IGF-1) signaling, which manifests as severe muscle wasting (6–15). Recently, skeletal muscle mitochondrial and redox abnormalities have emerged as potential causal factors driving the skeletal myopathy in CKD (2, 16–28); however, the mechanisms governing metabolic changes are not fully understood.

The accumulation of uremic metabolites and solutes is considered a hallmark of CKD and has deleterious effects to multiple tissues (29–32). Indoxyl sulfate (IS), a well-known uremic metabolite, has been shown to impair mitochondrial respiration, increase oxidative stress, and result in muscle atrophy in mice with normal kidney function (26, 27, 33). Kynurenines are another class of uremic metabolites that accumulate in patients with CKD and have been associated with low walking speed, grip strength, and frailty in non-CKD adults (34–36). Both indoles and kynurenines are derived from tryptophan (Tryp) catabolism and, interestingly, are ligands for the aryl hydrocarbon receptor (AHR) (37, 38), a ubiquitously expressed ligand-activated transcription factor involved in xenobiotic metabolism of both endogenous and exogenous molecules (39, 40). Chronic AHR activation, primarily studied in the context of exposure to dioxin, is toxic in the liver, reproductive organs, immune system, and central nervous system (39, 41–43). These toxic effects have been associated with disruption of circadian rhythm, metabolic syndrome, and type II diabetes (42, 44, 45). Elevated levels of AHR activation have been identified in the blood of patients with CKD (46) and in several tissues of rodents with CKD (47). In skeletal muscle, recent work has shown that AHR activation phenocopies the skeletal myopathy caused by tobacco smoking (48) and contributes to worsened myopathy outcomes in the context of limb ischemia (49). Based on the prior evidence, this study aimed to test whether AHR activation links the accumulation of uremic metabolites to muscle dysfunction in CKD.

## Results

### AHR activation is present in skeletal muscle of patients and rodents with CKD.

**Figure 1:**
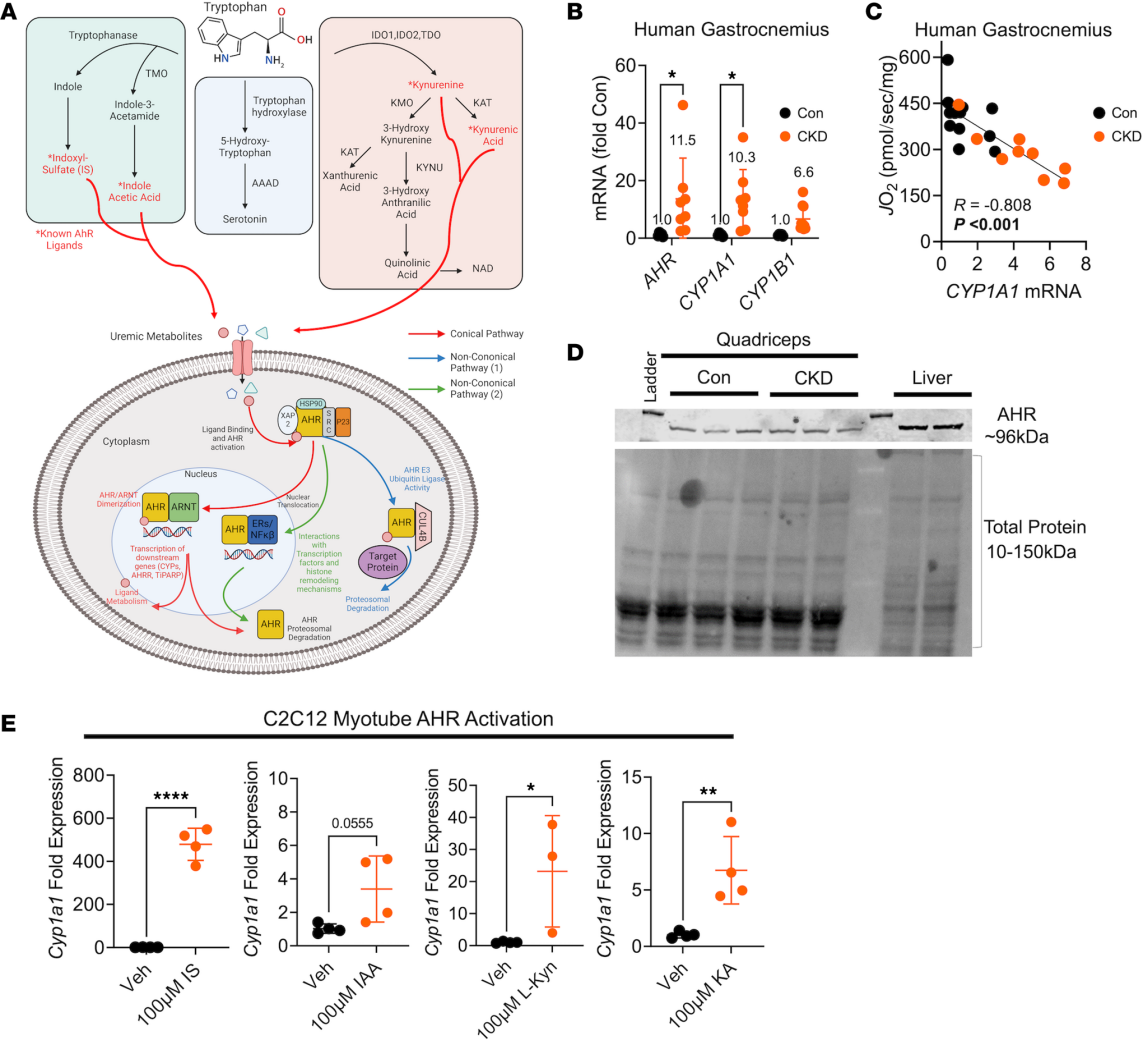
AHR activation is present in CKD skeletal muscle and associates with mitochondrial respiratory function. (**A**) Graphical depiction of tryptophan metabolism and the AHR signaling pathway. (**B**) qPCR quantification of *AHR*, *CYP1A1*, and *CYP1B1* mRNA signaling in gastrocnemius muscle biopsies from patients without (*n* = 5) and with CKD (*n* = 8–10). (**C**) Relationship between muscle mitochondrial oxygen consumption (*J*O_2_) and *CYP1A1* in patients with and without CKD. (**D**) Immunoblotting of the AHR protein in skeletal muscle of mice. (**E**) qPCR quantification of *Cyp1a1* mRNA levels in C_2_C_12_ myotubes treated with tryptophan-derived uremic metabolites indoxyl sulfate (IS), indole-3-acetic acid (IAA), L-kynurenine (L-Kyn), and kynurenic acid (KA) (*n* = 3–4 biological replicates/group). Statistical analyses performed using 2-tailed Student’s *t* test. Data are shown as mean ± SD. *P < 0.05, **P < 0.01, and ****P < 0.0001.

Several uremic metabolites that accumulate in the serum of patients with CKD are derived from Tryp catabolism (50–53) (Figure 1A). To explore if the accumulation of Tryp-derived uremic metabolites results in AHR activation in skeletal muscle, we employed quantitative PCR (qPCR) to measure the mRNA expression of the *AHR* and downstream cytochrome P450 genes*, CYP1A1* and *CYP1B1*, in gastrocnemius muscle from participants with and without CKD. *AHR* and *CYP1A1* mRNA expression were increased ~11.5- and ~10.3-fold in muscle from patients with CKD when compared with controls (Figure 1B). *CYP1B1* was increased ~6.6-fold in CKD, but this was not statistically significant (*P* = 0.525) (Figure 1B). The expression of *CYP1A1* (a surrogate for AHR activation) had a significant inverse association with muscle mitochondrial respiration rates in permeabilized myofibers (Figure 1C). Immunoblotting performed on the quadriceps muscle of mice confirmed the presence of the AHR protein, although abundance was not affected by CKD and was lower than the liver (Figure 1D). Next, cultured murine (C_2_C_12_) myotubes treated with 100 μM of Tryp-derived uremic metabolites (IS, kynurenic acid [KA], L-kynurenine [L-Kyn], and indole-3-acetic acid [IAA]) displayed increases in *Cyp1a1* mRNA expression (Figure 1E). These data demonstrate that the AHR is expressed in human and mouse skeletal muscle and is activated in the context of CKD and by Tryp-derived uremic metabolites.

### Uremic metabolite accumulation drives skeletal muscle AHR activation in CKD and can be disrupted by muscle-specific AHR deletion.

**Figure 2:**
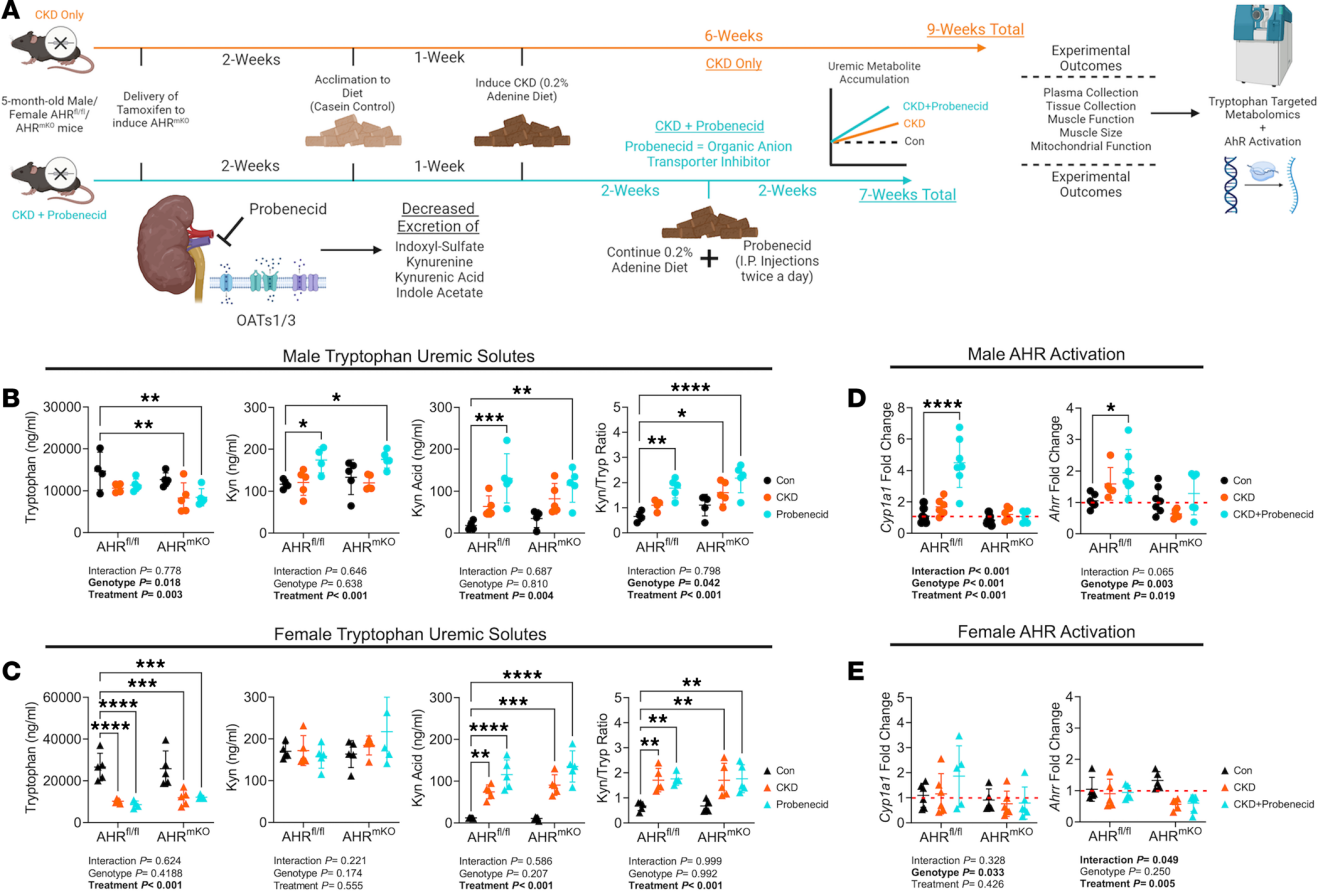
Uremic metabolite accumulation drives AHR activation in CKD muscle, which is abolished by muscle-specific AHR deletion. (**A**) Experimental treatment timeline. (**B**) Concentrations of tryptophan-derived uremic metabolites in plasma from male AHR^fl/fl^ and AHR^mKO^ mice without CKD, with CKD, and with CKD plus daily probenecid treatment (*n* = 4–5/group/genotype). (**C**) Concentrations of tryptophan-derived uremic metabolites in plasma from female AHR^fl/fl^ and AHR^mKO^ mice without CKD, with CKD, and with CKD plus daily probenecid treatment (*n* = 4–5/group/genotype). (**D**) qPCR quantification of *Cyp1a1* and *Ahrr* levels in skeletal muscle of male AHR^fl/fl^ and AHR^mKO^ mice without CKD, with CKD, and with CKD plus daily probenecid treatment (*n* = 5–7/group/genotype). (**E**) qPCR quantification of *Cyp1a1* and *Ahrr* levels in skeletal muscle of female AHR^fl/fl^ and AHR^mKO^ mice without CKD, with CKD, and with CKD plus daily probenecid treatment (*n* = 5–6/group/genotype). Statistical analyses performed using 2-way ANOVA with Dunnett’s post hoc testing for multiple comparisons. Data are shown as mean ± SD. *P < 0.05, **P < 0.01, ***P < 0.001, and ****P < 0.0001.

To determine if serum levels of uremic metabolites are responsible for AHR activation in skeletal muscle, we generated an inducible skeletal muscle–specific KO mouse (AHR^mKO^). Deletion of the AHR was confirmed in skeletal muscle by DNA recombination (Supplemental Figure 2A; supplemental material available online with this article; https://doi.org/10.1172/jci.insight.178372DS1) and by the ablation of AHR signaling (*Cyp1a1* mRNA expression) in muscle exposed to IS (Supplemental Figure 2B). Next, we explored the link between uremic metabolite accumulation and AHR activation using WT littermates (AHR^fl/fl^) and AHR^mKO^ mice fed either a casein control or adenine-supplemented diet (CKD), as well as CKD-mice treated twice daily with probenecid, an organic anion transporter inhibitor that has been shown to further increase uremic metabolite levels by preventing tubular secretion (54) (Figure 2A). L-Kyn, KA, and the L-Kyn/Tryp ratio (Kyn/Tryp) were all significantly elevated in probenecid treated male mice with CKD (Figure 2B). Interestingly, kynurenine concentrations remained unchanged in females while KA and Kyn/Tryp were significantly elevated in both CKD only and probenecid groups (Figure 2C). *Cyp1a1* and *Ahrr* (genes regulated by the AHR) were significantly increased in muscle from AHR^fl/fl^ male mice and unaffected in the AHR^mKO^ mice (Figure 2D). However, females elicited lower activation of AHR-dependent genes compared with males (Figure 2E). These sex-dependent effects appear to be independent of the severity of CKD, since both males and females displayed similar glomerular filtration rates (GFR) (Supplemental Figure 2C) and blood urea nitrogen levels (Supplemental Figure 2D).

### Deletion of the AHR disrupts uremia-induced mitochondrial OXPHOS dysfunction in skeletal muscle.

**Figure 3:**
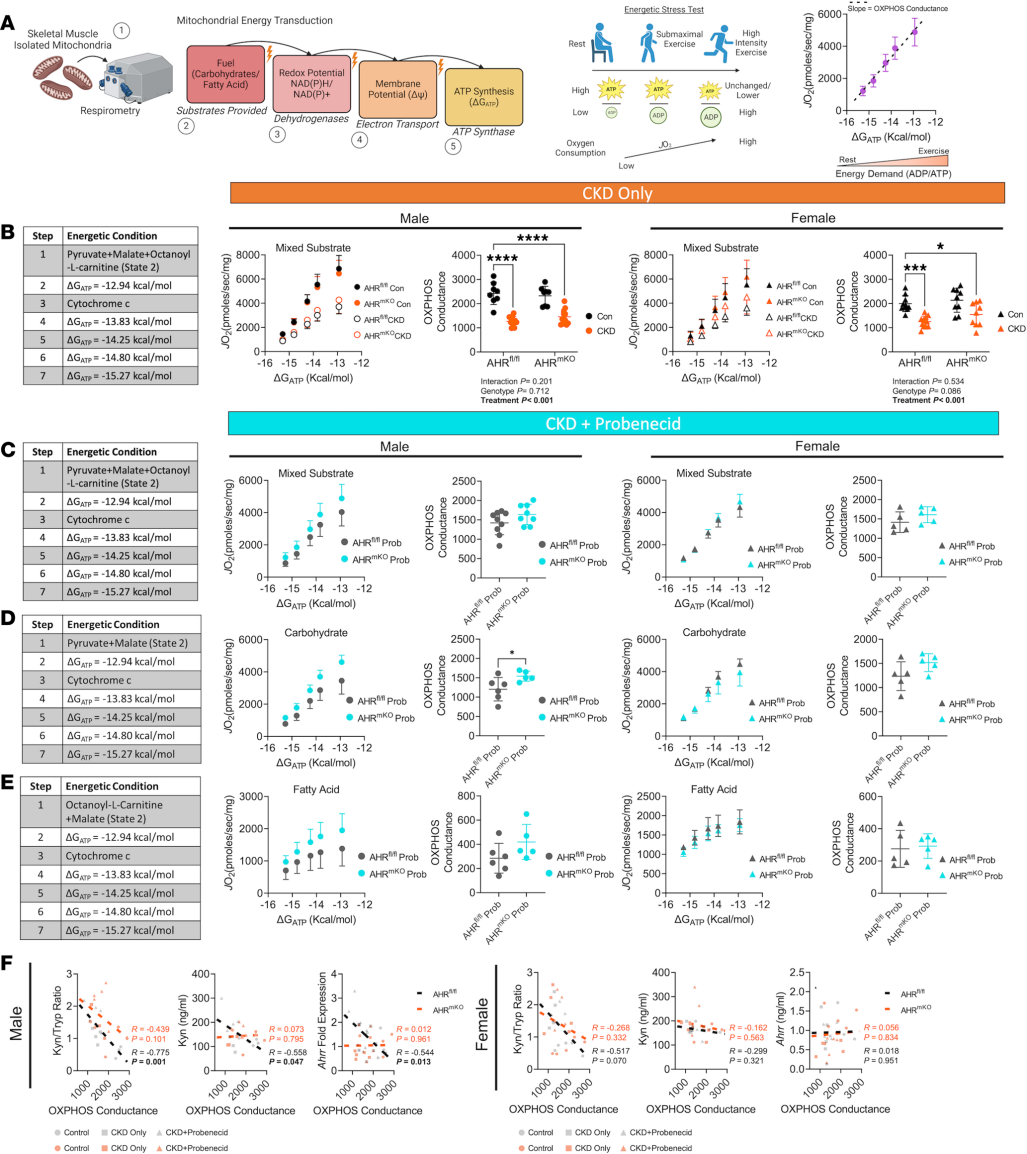
Muscle-specific AHR deletion improves mitochondrial OXPHOS with high tryptophan-derived uremic metabolite levels. (**A**) Graphical depiction of mitochondrial OXPHOS system and the use of a creatine kinase clamp to measure oxygen consumption (*J*O_2_) across physiologically relevant energetic demands (ΔG_ATP_). (**B**) Experimental conditions quantification *J*O_2_ at each level of ΔG_ATP_, as well as the OXPHOS conductance in male and female AHR^fl/fl^ and AHR^mKO^ mice with or without CKD (*n* = 8–12/group/genotype). (**C**–**E**) Experimental conditions and quantification *J*O_2_ at each level of ΔG_ATP_, as well as the OXPHOS conductance in male and female AHR^fl/fl^ and AHR^mKO^ mice with CKD plus daily probenecid treatment (*n* = 5–9/group/genotype) for mixed substrates (**C**), pyruvate/malate (**D**), and octanoylcarnitine/malate (**E**). (**F**) Pearson correlational analyses of quantified OXPHOS conductance (mixed substrates) and kynurenine to tryptophan ratio, kynurenine concentrations, and *Ahrr* mRNA in male and female AHR^fl/fl^ and AHR^mKO^ mice across control, CKD, and CKD plus probenecid daily. Data were analyzed by 2-way ANOVA with Dunnett’s post hoc testing for multiple comparisons in **B**. Two-tailed Student’s *t* test was performed in **C**–**E**. Data are shown as mean ± SD. *P < 0.05, ***P < 0.001, and ****P < 0.0001.

Next, we sought to determine if the significant association between *CYP1A1* expression levels and mitochondrial respiratory function observed in skeletal muscle from patients with and without CKD (Figure 1C) was mediated by the AHR. Mitochondria were isolated from the muscle of AHR^fl/fl^ and AHR^mKO^ mice, and respirometry was performed using a creatine kinase (CK) clamp to titrate the extra mitochondrial ATP/ADP ratio (ΔG_ATP_, a representation of cellular energy demand). The relationship between ΔG_ATP_ and oxygen consumption (*J*O_2_) represents the conductance through the mitochondrial OXPHOS system (Figure 3A). Using a mixture of carbohydrate and fatty acid to fuel mitochondria, *J*O_2_, and OXPHOS conductance was significantly decreased in mice with CKD (Figure 3B). However, deletion of the AHR did not significantly improve OXPHOS in CKD mice (Figure 3B). When probenecid was administered to mice with CKD to increase uremic metabolite levels and AHR activation in skeletal muscle further, deletion of the AHR was found to have sex-dependent and fuel source–dependent effects on muscle mitochondrial OXPHOS. Under these conditions, AHR^mKO^ failed to protect females from OXPHOS impairment when mitochondria were fueled by a mixture of carbohydrates and fatty acid (Figure 3C), consistent with the results in CKD mice without probenecid treatment. However, when mitochondria were energized with carbohydrates (pyruvate and malate), male AHR^mKO^ mice had significantly higher OXPHOS conductance compared with AHR^fl/fl^ littermates (*P* = 0.045, Figure 3D). No significant effect of AHR^mKO^ was observed when mitochondria were fueled only with the medium chain fatty acid octanoylcarnitine in males (Figure 3E) or in any condition in female mice (Figure 3, C–E).

Interestingly, elevated mRNA expression of pyruvate dehydrogenase kinase 4 (*Pdk4*), a negative regulator of pyruvate metabolism, was upregulated in male AHR^fl/fl^ mice with CKD + probenecid but not AHR^mKO^ mice (Supplemental Figure 3A), and this could explain the protection of OXPHOS observed in AHR^mKO^ mice when pyruvate is the primary carbon source. Mitochondrial H_2_O_2_ production was unaffected by the presence of CKD or the deletion of the AHR in either sex (Supplemental Figure 3B). Additionally, probenecid treatment alone did not have an effect on OXPHOS conductance in skeletal muscle mitochondria (Supplemental Figure 4). We observed strong inverse correlations between uremic metabolite levels (Kyn/Trp ratio and Kyn concentration) or AHR activation (*Ahrr* expression) and OXPHOS conductance in male AHR^fl/fl^ mice but not in females (Figure 3F). Interestingly, those relationships were abolished in AHR^mKO^ male mice. These findings are in agreement with the observed relationship between AHR activation and *J*O_2_ in human CKD skeletal muscle (Figure 1C) and previous work in non-CKD rodents exposed to elevated kynurenines (55). While CKD decreased muscle mass, myofiber size/area, grip strength, and isometric contractile performance, deletion of the AHR did not attenuate these changes in either sex (Supplemental Figures 5 and 6).

### Muscle-specific knockdown of the AHR in CKD mice expressing a high-affinity AHR allele improves mitochondrial OXPHOS.

**Figure 4:**
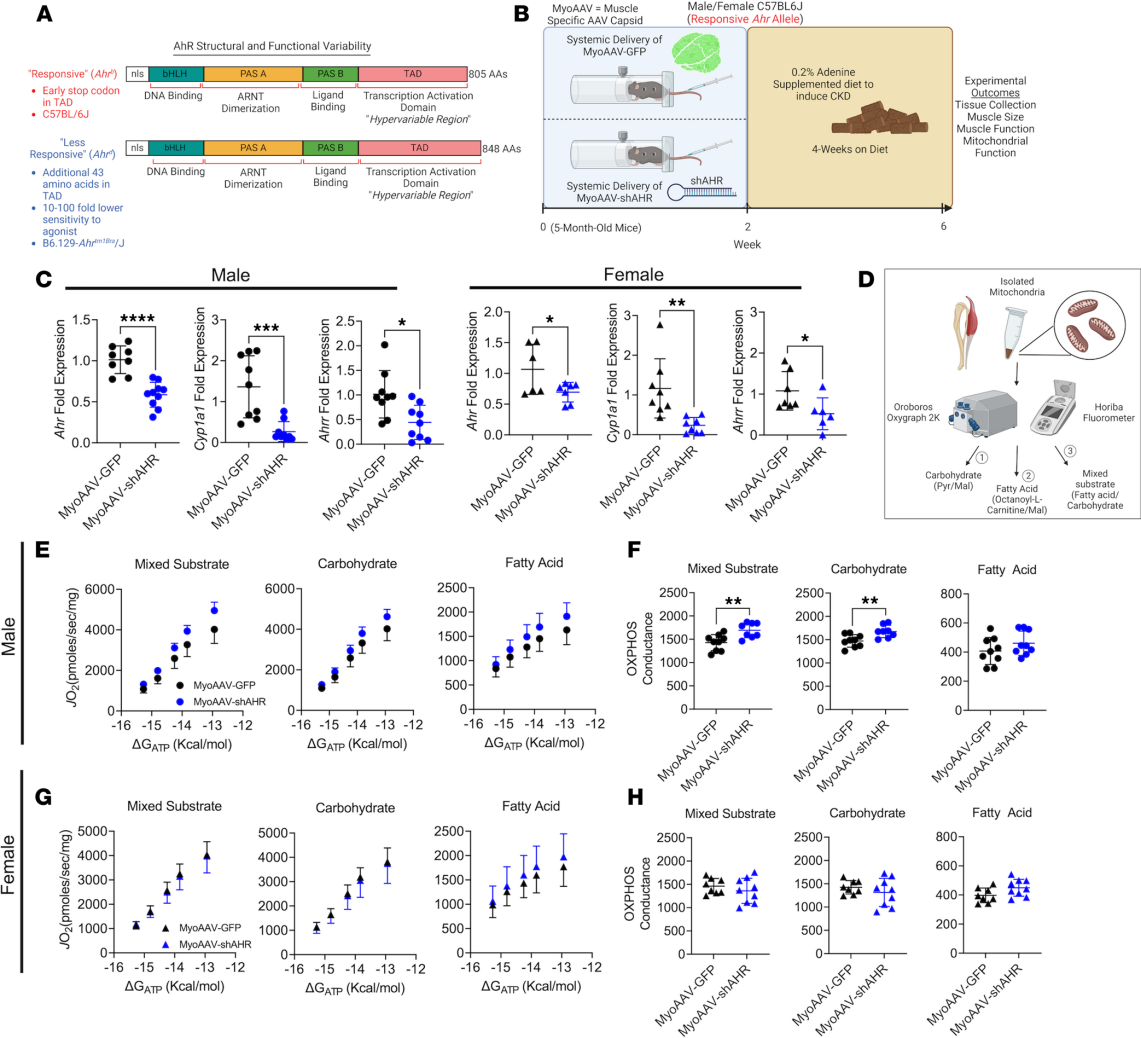
Muscle-specific AHR knockdown improves mitochondrial OXPHOS in mice harboring the high-affinity AHR allele. (**A**) Graphical depiction of polymorphisms in the AHR that confer differences in ligand affinity. (**B**) Experimental timeline of delivery of MyoAAV-GFP or MyoAAV-shAHR in high-affinity C57BL/6J mice with CKD. (**C**) qPCR validation of *Ahr* knockdown and subsequent reduction in *Cyp1a1* and *Ahrr* mRNA induction in MyoAAV-shAHR mice (*n* = 6–10/group). (**D**) Graphical depiction of analytical approach for mitochondrial OXPHOS assessments. (**E**) Relationship between *J*O_2_ and ΔG_ATP_ in isolated mitochondria from the gastrocnemius muscle in different substrate conditions in male mice with CKD (*n* = 8–9/group). (**F**) Quantification of OXPHOS conductance in male mice (*n* = 8–9/group). (**G**) Relationship between *J*O_2_ and ΔG_ATP_ in isolated mitochondria from the gastrocnemius muscle in different substrate conditions in female mice with CKD (*n* = 8–9/group). (**H**) Quantification of OXPHOS conductance in female mice (*n* = 8–9/group). Statistical analyses were performed using 2-tailed Student’s *t* test. Data are shown as mean ± SD. *P < 0.05, **P < 0.01, ***P < 0.001, and ****P < 0.0001.

While the AHR is well conserved across species, naturally occurring polymorphisms in the sequence exist and confer differences in the affinity for ligands (56–58). The AHR^fl/fl^ mice used to generate the AHR^mKO^ mice herein were derived from 129-SvJ embryonic stem cells, which harbor a low-affinity AHR^d^ allele that exhibits 10- to 100-fold lower sensitivity to xenobiotic ligands when compared with mice with the high-affinity AHR^b^ found in C57BL/6J mice (56) (Figure 4A). Thus, we examined if knockdown of the AHR in muscle of C57BL/6J mice that harbor the high-affinity AHR allele would attenuate muscle pathology in CKD. Muscle-specific knockdown of the AHR was induced by systemic delivery of muscle-trophic adeno-associated virus (MyoAAV) (59) encoding a short hairpin RNA sequence targeting the AHR (shAHR) to mice with CKD (Figure 4B). Compared with CKD mice that received MyoAAV-GFP, *Ahr*, *Cyp1a1*, and *Ahrr* mRNA levels were significantly reduced in the skeletal muscle of CKD mice that received MyoAAV-shAHR (Figure 4C). No differences were observed in AHR mRNA levels in the liver (Supplemental Figure 7A). Examination of mitochondrial function in the gastrocnemius muscle (Figure 4D) revealed significantly higher mitochondrial OXPHOS in male CKD mice that received MyoAAV-shAHR when mitochondria were fueled by a mixture of carbohydrate and fatty acid substrates as well as when they were fueled by carbohydrates only (both *P* < 0.01), but not when energized with octanoylcarnitine alone (Figure 4, E and F). Consistent with results from low-affinity AHR^mKO^ mice, MyoAAV-shAHR had no effect on mitochondrial OXPHOS in female mice (Figure 4, G and H). Mitochondrial H_2_O_2_ production, muscle mass, and muscle contractile function were not different between treatment groups (Supplemental Figure 7).

### Skeletal muscle–specific expression of a constitutively active AHR (CAAHR) in mice with normal kidney function impairs mitochondrial energetics.

**Figure 5:**
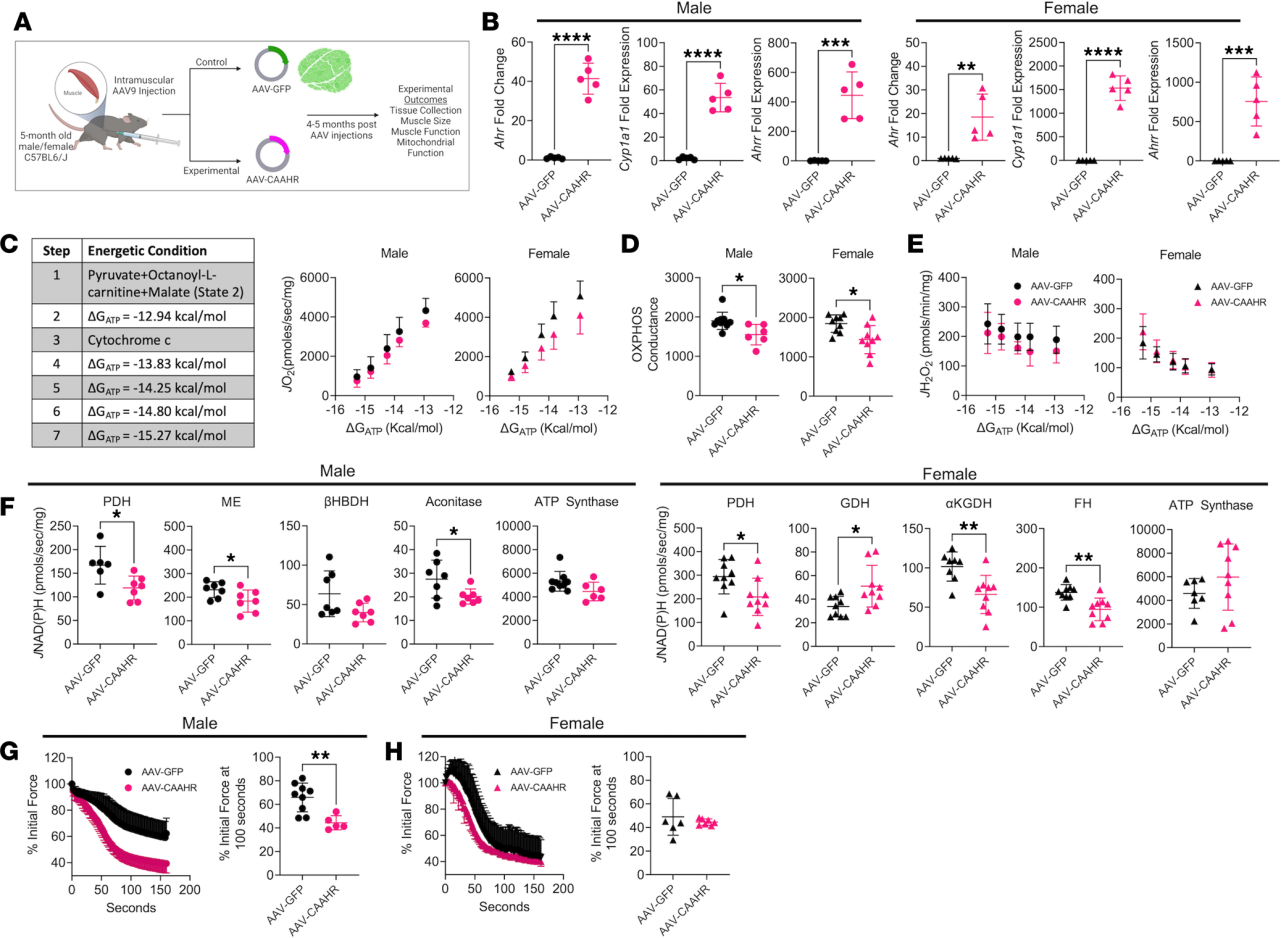
Ectopic expression of a constitutively active AHR impairs muscle mitochondrial OXPHOS in mice with normal kidney function. (**A**) Experimental design for muscle-specific delivery of mutant constitutively active AHR (CAAHR). (**B**) qPCR of *Ahr*, *Cyp1a1*, and *Ahrr* in male and female mice treated with AAV-GFP and AAV-CAAHR (*n* = 5/group). (**C**) Substrate conditions and quantification of the relationship between *J*O_2_ and ΔG_ATP_ in male and female mice treated with AAV-GFP or AAV-CAAHR (*n* = 6–10/group). (**D**) OXPHOS conductance in male and female mice (*n* = 6–10/group). (**E**) Mitochondrial *J*H_2_O_2_ and ΔG_ATP_ in male and female mice (*n* = 6–10/group). (**F**) Quantification of mitochondrial matrix dehydrogenase enzyme activity in male and female mice (*n* = 6–9/group). (**G** and **H**) Analysis of extensor digitorum longus muscle fatigue in male and female mice (*n* = 5–9/group). Data analyzed using 2-tailed Student’s *t* test. Data are shown as mean ± SD. *P < 0.05, **P < 0.01, ***P < 0.001, and ****P < 0.0001.

To isolate the role of AHR activation from the complex milieu of renal insufficiency, we generated a mutant AHR that displays constitutive transcriptional activity in the absence of ligands (60) (termed CAAHR herein). The CAAHR, or a GFP control, was delivered to mice with normal renal function using AAV9 and the skeletal muscle–specific promoter (human skeletal actin [HSA]; *ACTA1* gene) (Figure 5A). Constitutive AHR activation was confirmed via *Ahr*, *Cyp1a1*, and *Ahrr* mRNA expression (Figure 5B). Interestingly, *Cyp1a1* expression was higher in females than males treated with AAV-CAAHR, but this was not caused by sex-dependent differences in *Ahr* repression, since *Ahrr* expression was similar between males and females (Figure 5B). Skeletal muscle OXPHOS function was significantly lower in AAV-CAAHR mice compared with AAV-GFP mice, regardless of sex (Figure 5, C and D). Mitochondrial H_2_O_2_ production was unaffected by AAV-CAAHR treatment (Figure 5E). To explore the mechanisms underlying OXPHOS dysfunction coincident with AHR activation, we assayed several matrix dehydrogenase enzymes. AAV-CAAHR reduced the activity of pyruvate dehydrogenase (PDH), malic enzyme (ME), and aconitase in males (Figure 5F). In females, AAV-CAAHR decreased the activity of PDH, α-ketoglutarate dehydrogenase (AKGDH), and fumarate hydratase (FH), but it increased glutamate dehydrogenase (GDH) activity (Figure 5F). Additional dehydrogenase assays that were unaffected by CAAHR are shown in Supplemental Figure 8. Unexpectedly, AAV-CAAHR hastened muscle fatigue in male mice only (Figure 5, G and H) but did not affect muscle mass or strength in either sex (Supplemental Figure 8).

### Ahr activation drives Pdk4-induced phosphorylation of the PDH enzyme.

**Figure 6:**
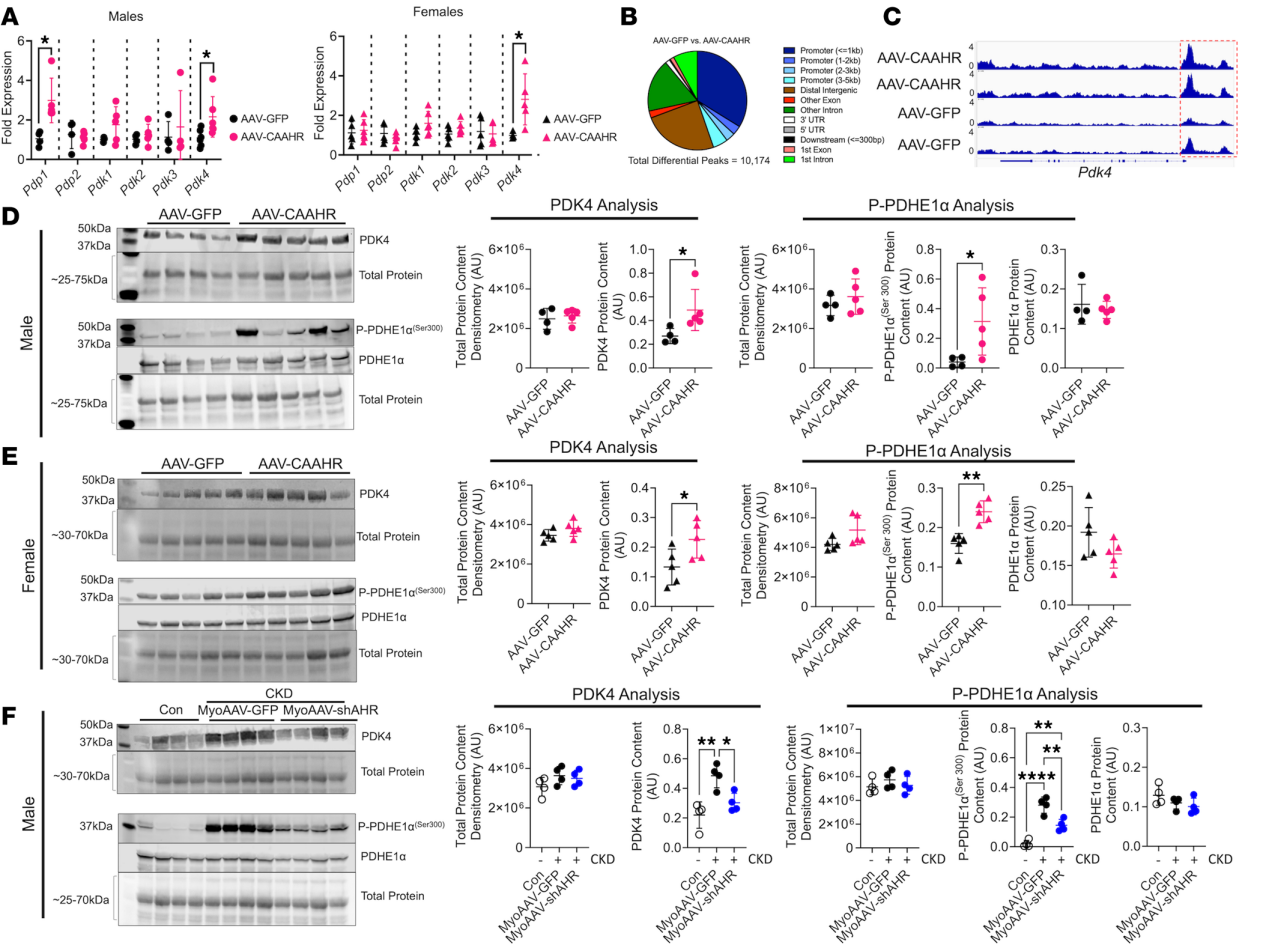
AHR activation increased PDK4 expression and PDH phosphorylation. (**A**) qPCR of *Pdp1*, *Pdp2*, *Pdk1*, *Pdk2*, *Pdk3*, and *Pdk4* in male and female mice treated with AAV-GFP and AAV-CAAHR (*n* = 5–6/group). (**B**) Peak annotation pie charts for ATAC-Seq peaks in AAV-GFP versus AAV-CAAHR muscles (*n* = 3/group). (**C**) IGV snapshots of the *Pdk4* gene showing chromatin accessibility, with the red-dashed box highlighting the promoter region. (**D**) Western blotting of PDK4, phosphorylated PDHE1α^Ser300^, and total PDHE1α protein expression in male AAV-GFP or AAV-CAAHR gastrocnemius muscle (*n* = 4–5/group). (**E**) Western blotting of PDK4, phosphorylated PDHE1α^Ser300^, and total PDHE1α protein expression in female AAV-GFP or AAV-CAAHR gastrocnemius muscle (*n* = 5/group). (**F**) Western blotting of PDK4, phosphorylated PDHE1α^Ser300^, and total PDHE1α protein expression in male control, CKD MyoAAV-GFP, and CKD MyoAAV-shAHR gastrocnemius muscle (*n* = 4/group). Data in **A**, **D**, and **E** were analyzed using 2-tailed Student’s *t* test. Data in **F** were analyzed using 1-way ANOVA with Tukey’s post hoc test. *P < 0.05, **P < 0.01, and ****P < 0.0001.

Since OXPHOS function was altered by AHR activation primarily when pyruvate was supplied as a fuel source, we explored if posttranslational modification of the PDH enzyme could be linked to AHR activation. The activity of PDH is regulated by its phosphorylation status, where PDH kinases (PDKs) decrease activity and PDH phosphatases (PDPs) increase activity. qPCR for PDK and PDP genes in skeletal muscle revealed a significant increase in the mRNA expression of *Pdk4* in both male and female mice treated with AAV-CAAHR, while other PDK isoforms (*Pdk1*, *Pdk2*, *Pdk3*) were unaltered (Figure 6A). Male mice treated with AAV-CAAHR had increased *Pdp1* expression (Figure 6A), suggesting a possible compensatory response to elevated *Pdk4*. Using assay for transposase-accessible chromatin sequencing (ATAC-Seq) to explore chromatin accessibility, there were more than 10,000 differentially accessible peaks between AAV-CAAHR and AAV-GFP muscle (Figure 6B). Accessibility to the promoter region of *Pdk4* was noticeably different between AAV-CAAHR and AAV-GFP muscle (Figure 6C).

**Figure 7:**
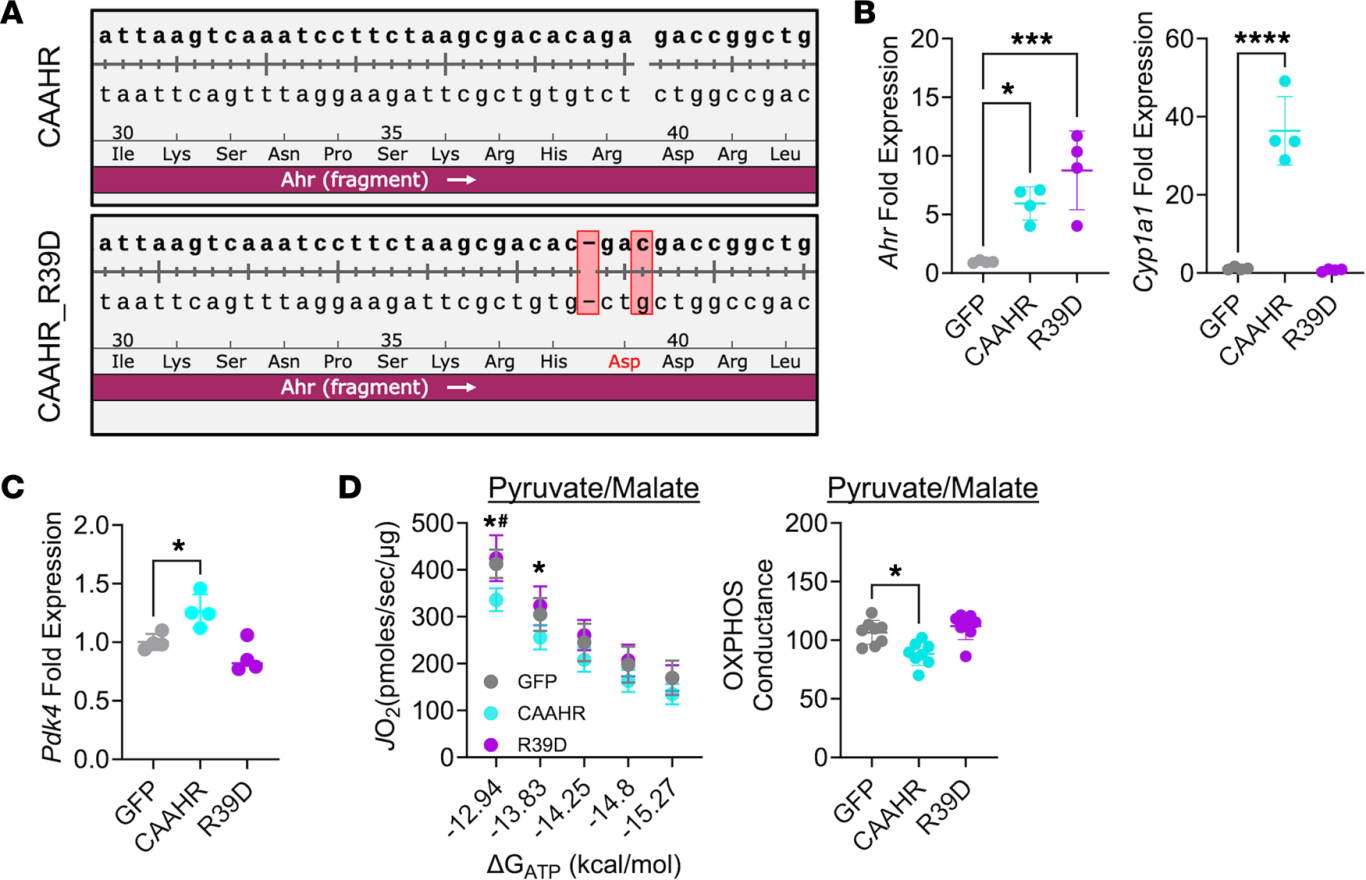
Expression of a transcriptionally inept CAAHR abolishes Pdk4 expression and pyruvate-supported OXPHOS impairment in C_2_C_12_ muscle cells. (**A**) Sequencing results demonstrating the introduction of point mutation that converted arginine-39 to aspartate (R39D). (**B**) qPCR validation of the overexpression of *Ahr* and lack of transcriptional activity (*Cyp1a1*) in the R39D mutant. A GFP control plasmid was also tested (*n* = 4/group). (**C**) *Pdk4* mRNA expression (fold GFP) (*n* = 4/group). (**D**) Pyruvate supported respiration in muscle cells and quantified OXPHOS conductance (*n* = 8/group). Data are shown as mean ± SD. Data were analyzed using 1-way ANOVA with Tukey’s post hoc test. *P < 0.05, ***P < 0.001, and ****P < 0.0001. ^#^*P* < 0.05 indicates CAAHR versus R39D.

Next, we performed immunoblotting experiments to examine PDK4 protein abundance and the phosphorylation status of the PDH enzyme (uncropped blots shown in Supplemental Figure 9). In male and female mice treated with AAV-CAAHR, PDK4 protein abundance and phosphorylation of PDHE1α at serine 300 were significantly increased compared with AAV-GFP–treated mice (Figure 6, D and E). No changes in total PDHE1α protein content were observed in either sex (Figure 6, D and E). Additionally, we performed experiments on non-CKD control mice, mice with CKD treated with MyoAAV-GFP, and mice with CKD treated with MyoAAV-shAHR (only male analyses are shown due to no improvements found in OXPHOS of female MyoAAV-shAHR mice; Figure 4). MyoAAV-GFP mice with CKD had elevated PDK4 protein abundance and increased phosphorylation of PDHE1α at serine 300 when compared with non-CKD control mice (Figure 6F). MyoAAV-shAHR treatment significantly decreased the abundance of both the PDK4 protein and the phosphorylation of PDHE1α at serine 300 (Figure 6F). Using cultured muscle cells, IS and L-Kyn treatment were also found to increase *Pdk4* mRNA expression and the phosphorylation of PDHE1α at serine 300 (Supplemental Figure 10). To confirm transcription regulation of *Pdk4* by the AHR, we generated a transcriptionally inept CAAHR by mutating the 39th amino acid from arginine to aspartate (R39D), which dramatically reduces DNA binding affinity (61) (Figure 7A). Whereas expression of the CAAHR and R39D mutant both increase *Ahr* mRNA levels equally, *Cyp1a1* expression was only increased in the CAAHR-treated muscle cells (Figure 7B). *Pdk4* mRNA levels were significantly increased in muscle cells treated with the CAAHR, whereas the R39D mutant and GFP-treated muscle cells had similar *Pdk4* expression (Figure 7C). Compared with GFP- or R39D-treated muscle cells, CAAHR-treated cells had significantly impaired pyruvate-supported OXPHOS (Figure 7D).

## Discussion

A progressive skeletal myopathy has been established in patients with CKD and contributes to symptoms of exercise intolerance and lower quality of life. Whereas the pathways driving muscle wasting/atrophy in CKD have been well described (10, 62), less is understood about the metabolic insufficiency observed in skeletal muscle of these patients (4, 18, 20, 63, 64). In this study, we identified AHR activation in the skeletal muscle of patients and mice with CKD. Skeletal muscle–specific deletion of the AHR in mice with CKD and elevated Tryp-derived uremic metabolites significantly improved mitochondrial OXPHOS in male mice only, and these improvements were greatest when mitochondria were fueled by pyruvate rather than fatty acids. Mechanistically, AHR activation in muscle resulted in increased PDK4 expression (mRNA and protein) and subsequent phosphorylation of the PDH enzyme causing impaired enzyme activity.

CKD is a multifactorial disease that complicates investigations to understand skeletal muscle pathology. Contributing factors include metabolic acidosis, chronic inflammation, overactivation of renin angiotensin signaling, oxidative stress, and retention of uremic metabolites, often described as “toxins.” The accumulation of Tryp-derived uremic metabolites including IS, IAA, L-Kyn, and KA have been associated with disease severity and mortality rates in patients with CKD (65–68). Treatment with AST-120, an orally administered spherical carbon adsorbent that lowers IS levels in systemic circulation (69), was reported to improve exercise capacity and muscle mitochondrial biogenesis in mice with CKD (70). However, in a randomized controlled trial with patients with CKD, AST-120 failed to significantly improve walking speed, grip strength, muscle mass, or perceived quality of life (71). This brings to question whether other uremic metabolites are contributing to muscle pathology in CKD. Kynurenines have been associated with chronic inflammation and uremic symptoms in patients with CKD (66), and mice with elevated circulating kynurenine display impaired muscle OXPHOS function (55). Notably, kynurenine and KA levels increase significantly with respect to CKD severity and are incompletely removed from the blood by hemodialysis treatment (66). Moreover, prolonged PCr recovery in skeletal muscle of patients with CKD (a marker of in vivo mitochondrial dysfunction) was found to associate with eGFR, occurred prior to initiation of hemodialysis, and was lowest in patients receiving hemodialysis treatment (2). Thus, the progressive accumulation of uremic metabolites, especially ones that may be poorly filtered by conventional dialysis membranes, may be significant contributors to the progressive decline of mitochondrial health observed in patients with CKD.

Indoles and kynurenines are known ligands of the AHR (37, 38, 72), whose prolonged activation has been associated with the development of metabolic syndrome (44, 45), disruption of circadian rhythms (73), altered glucose and lipid metabolism (45, 74, 75), and mitochondrial respiratory impairments (76–78). To date, only 3 studies have investigated the role of the AHR in skeletal muscle (26, 48, 49), although previous studies have reported AHR activation in the blood of patients with CKD (46, 79). In this study, skeletal muscle–specific AHR deletion improved mitochondrial OXPHOS function in CKD mice only in combination with probenecid treatment to further elevate uremic metabolites and AHR activation. However, it is important to note that several naturally occurring AHR polymorphisms occur in mice and that the AHR^mKO^ mice used in this study harbor a less-sensitive Ahr^d^ allele, as compared with the Ahr^b1^ allele found in C57BL/6J mice, because they were generated using 129-SvJ embryonic stem cells (80). Thus, they have lower levels of AHR activation for a given dosage of ligand compared with the AHR allele found in C57BL/6J mice. To address this issue, we performed several experiments. First, AHR^mKO^ and littermates with CKD were treated with probenecid, an organic anion transporter inhibitor that decreases the kidney’s ability to eliminate uremic toxins (54). Probenecid was found to enhance serum uremic metabolite levels and muscle AHR activation (*Cyp1a1* and *Ahrr* mRNA expression), particularly in male mice (Figure 2). Consequently, higher levels of AHR activation caused by treatment with probenecid revealed a significant improvement in mitochondrial OXPHOS in male mice but not female mice (Figure 3). Next, we performed experiments in the C57BL/6J mouse that expresses the high affinity Ahr^b1^ allele by employing genetic knockdown (MyoAAV-shAHR) in CKD mice. In each of these, limiting AHR activation in CKD was found to promote improvements in mitochondrial OXPHOS with carbohydrate fuels in male but not female mice. Whether or not a progressive AHR activation occurs across increasing stages of CKD in patients remains to be explored. However, it is intriguing that 2 studies in patients with CKD have reported stepwise impairment of muscle mitochondrial function with increasing CKD severity. Bittel et al. (81) reported that carbohydrate-supported mitochondrial respiration (measured ex vivo) decreased with CKD severity. Similarly, in vivo phosphorus magnetic resonance spectroscopy analyses of muscle energetics performed by Gamboa et al. (2) showed a progressive increase in the time constant for phosphocreatine (PCr) resynthesis (an index of lower muscle oxidative capacity) across tertiles of eGFR. While more experimentation is necessary, these observations align with our observation that AHR activation is inversely correlated with muscle mitochondrial respiration.

While the mechanisms underlying the fact that AHR deletion and knockdown improved OXPHOS in male mice only are unknown, reports of sexual dimorphism in AHR biology have been reported. For example, differences in the response to 2, 3, 7, 8-tetrachlorodibenzodioxin (TCDD, a potent AHR agonist) treatment have been reported in the livers of male and female mice (82). Furthermore, it has been reported that the ligand activated AHR complex can physically associate with the estrogen receptor as well as the androgen receptor and alter sex hormone signaling (83). AHR activation has also been shown to promote proteasomal degradation of the estrogen receptor through the cullin 4B ubiquitin ligase pathway (84) and alter sex hormone secretion (85). It is unknown if there are sex-dependent differences in AHR biology in patients with CKD or regarding muscle mitochondrial function, although several studies investigating muscle energetics in patients with CKD have included both male and female patients and sex differences were not specifically described (2, 4, 18).

Enhanced mitochondrial OXPHOS function in male mice with AHR deletion or knockdown was present only when pyruvate was the primary fuel source. This was similar to a recent study exploring ischemic myopathy in the context of CKD (49). Additionally, C_2_C_12_ myotubes treated with uremic serum from rodents exhibited lower OXPHOS function in the presence of glucose but not when fueled by fatty acids (86). Regarding potential mechanisms by which AHR may impair muscle mitochondrial OXPHOS, we found that PDH activity was significantly lower in both male and female mice that received AAV-CAAHR treatment. This is noteworthy because several studies have shown that uremic metabolites alone (25), as well as CKD (22), can impair matrix dehydrogenase activity. Protein and mRNA analysis of mouse muscle from both CKD animals and those with ectopic CAAHR expression confirmed that AHR activation resulted in significant increases in the expression of PDK4, a negative regulator of the PDH enzyme, as well as phosphorylation of the PDH enzyme (Figure 6). Further experimentation in cultured muscle cells uncovered increased PDH phosphorylation following treatment with AHR ligands IS and L-Kyn (Supplemental Figure 10). In support of these findings, patients with CKD have been reported to display decreased PDH activity and upregulated PDK4 expression in skeletal muscle (87). Taken together, these findings establish a uremic metabolite/Ahr/Pdk4 axis as a mechanism contributing to skeletal muscle mitochondrial OXPHOS impairment in CKD.

Incongruent with our hypothesis, deletion of the AHR in skeletal muscle did not improve muscle size or function in mice with CKD. This contrasts with our recent study on the ischemic myopathy with CKD (49). A possible explanation for lack of agreement likely stems from the hypoxic/ischemic microenvironment, especially considering that the AHR’s transcriptional fidelity requires dimerization with the AHR nuclear translocator (ARNT), which is also known as hypoxia inducible factor 1-beta (HIF1b). The lack of improvement in muscle function or size with AHR deletion observed herein may be attributed to non–AHR-dependent effects of CKD and uremic metabolites. For example, IS was found to increase reactive oxygen species (ROS) production via activation of NADPH oxidases in cultured muscle cells (26). This increase in NADPH oxidase activity might initiate ROS-dependent atrophy pathways (88, 89), which are elevated in CKD muscle. Other contributing factors may include metabolic acidosis, chronic inflammation, or overactivation of renin angiotensin signaling in the CKD condition that do not involve the AHR.

The current study is not without limitations. First, due to limited specimen size in muscle biopsy tissue from participants, it was not possible to perform comprehensive assessments on skeletal muscle mitochondrial function as done in the animal models. Second, although mice used were fully mature and females were ovariectomized to better mimic the postmenopausal state of most female patients with CKD (90), the mice used in this study were relatively young despite age being a significant risk factor for CKD. Because the OXPHOS assessments employed require harvesting muscle tissue, these analyses were terminal, and repeated temporal assessments of mitochondrial OXPHOS were not possible. Thus, we could not establish whether the mitochondrial OXPHOS impairment (secondary to AHR activation) leads to muscle atrophy or contractile dysfunction with longer durations of AHR activation. All experiments involving rodents with CKD were performed on mice fed an adenine-supplemented diet, whereas other studies have employed surgical models of CKD (5/6 nephrectomy) (91, 92). We have shown that adenine and 5/6 nephrectomy models have similar levels of uremic metabolites, muscle atrophy, and mitochondrial dysfunction (24). Regarding uremic metabolites, it is worth noting that there may be differences in the relative abundance of AHR ligands in the adenine model compared with patients with CKD, although larger and more comprehensive quantification is necessary to fully assess these differences. Additionally, our metabolite analysis herein did not include quantification of indoles, although we have previously reported their increase in mice fed adenine diet to induce CKD (22, 24). Probenecid, a drug that reduces uric acid levels and is used to treat gout, was employed to elevate uremic metabolites levels as done previously (54), with the goal of increasing muscle AHR activation. Adenine is a purine base that can be converted to uric acid by xanthine oxidase, and the combination of adenine feeding and probenecid could affect the degree of renal impairment in our experiments, although blood urea nitrogen levels were similar in CKD and CKD + probenecid mice (Supplemental Figure 2). The degree of kidney injury with adenine feeding may be related to uric acid levels, as inhibition of xanthine oxidase attenuated kidney injury in this model (93). Nonetheless, it is important to consider any potential effects this combination could have because hyperuricemia occurs in patients with CKD and associates with mortality (94), and uric acid release occurs in atrophying muscle (95).

Collectively, the findings herein establish a Tryp-derived uremic metabolite/AHR/Pdk4 axis as a critical regulator of skeletal muscle mitochondrial function in CKD, when fueled by pyruvate, and provide evidence that interventions that disrupt this axis can improve muscle mitochondrial function.

## Methods

### Sex as a biological variable.

Our study examined male and female animals, and sex-dimorphic effects are reported. Participants included both male and female individuals (self-identified), but the sample size was not powered to detect differences in sex.

### Participants.

Muscle specimens of the gastrocnemius were collected from adult control participants with normal kidney function and patients with CKD via percutaneous muscle biopsy using sterile procedures (49, 96). The physical and clinical characteristics of these participants are shown in Supplemental Table 1. All participants in this study were free from peripheral vascular disease and distinct from our prior study on the role of the AHR in peripheral artery disease (49). Non-CKD adult controls and patients with CKD were recruited from the UF Health Shand’s Hospital or Malcom Randall VA Medical Center, Gainesville, Florida, USA. Inclusion criteria for patients with CKD included an eGFR between 15 and 45 mL/min/(1.73 × m^2^) for at least 3 months that were not on hemodialysis. Inclusion criteria for non-CKD adult controls was an eGFR greater than 80 mL/min/(1.73 × m^2^). eGFR was calculated using the CKD-EPI Creatinine equation (2021) (97). Exclusion criteria for both groups included being an active smoker (must be tobacco free for > 6 months), due to tobacco smoke containing AHR ligands (98). A portion of the muscle samples was cleaned and quickly snap frozen in liquid nitrogen. Another portion was immediately placed in ice-cold buffer X (50 mM K-MES, 7.23 mM K_2_EGTA, 2.77 mM CaK_2_EGTA, 20 mM imidazole, 20 mM taurine, 5.7 mM ATP, 14.3 mM PCr, and 6.56 mM MgCl_2_-6H_2_O, pH 7.1) for preparation of permeabilized fiber bundles (96, 99). Fiber bundles were mechanically separated using needle-tipped forceps under a dissecting scope and subsequently permeabilized with saponin (30 μg/mL) for 30 minutes at 4°C on a nutating mixer; they were then washed in ice-cold buffer Z (105 mM K-MES, 30 mM KCl, 1 mM EGTA, 10 mM K_2_HPO_4_, 5 mM MgCl_2_-6H_2_O, 0.5 mg/mL bovine serum albumin [BSA], pH 7.1) for 15 minutes until analysis. High-resolution O_2_ consumption measurements were conducted at 37°C in buffer Z (in mmol/L: 105 K-MES, 30 KCl, 1 EGTA, 10 K_2_HPO_4_, 5 MgCl_2_-6H_2_O; 0.5 mg/mL BSA, pH 7.1), supplemented with creatine monohydrate (5 mM), using the Oroboros O2K Oxygraph. Mitochondrial respiration was measured energizing the bundles with 5 mM pyruvate and 2.5 mM malate followed by the addition of 4 mM adenosine diphosphate (ADP) to stimulate maximal respiration. At the end of experiments, the bundles were retrieved, washed in distilled water, and lyophilized (Labconco), and the dry weight was obtained using a Mettler Toledo MX5 microbalance. Rates of *J*O_2_ were normalized to the bundle dry weight.

### Animals.

AHR conditional knockout mice (AHR^cKO^) with loxP sites flanking exon 2 of the AHR (AHR^tm3.1Bra/J^) were obtained from The Jackson Laboratory (stock no. 006203). AHR^cKO^ mice were bred with a tamoxifen-inducible skeletal muscle–specific Cre line (Tg[ACTA1-cre/Esr1*]2Kesr/J, The Jackson Laboratory, stock no. 025750) to generate skeletal muscle–specific inducible AHR-KO mice (AHR^mKO^). Female mice underwent bilateral ovariectomy (OVX) 14 days prior to inducing Cre-mediated DNA recombination. Deletion of the AHR was initiated at 5 months of age by i.p. injection of tamoxifen (MilliporeSigma, T5648) for 5 consecutive days (120 mg/kg). Littermate AHR-floxed mice without the Cre transgene (AHR^fl/fl^) that received the same tamoxifen dosing were used as controls. For AAV experiments, C57BL/6J mice (stock no. 000664) were obtained from The Jackson Laboratory at 5 months of age (*n* = 60 total mice). Female mice underwent OVX 14 days prior to delivery of AAV. All rodents were housed in a temperature- (22°C) and light-controlled (12-hour light/12-hour dark) room and maintained on standard chow diet (Envigo Teklad Global 18% Protein Rodent Diet 2918 irradiated pellet) with free access to food and water.

### Plasmid construction and AAV production/delivery.

AAV backbones were obtained from Cell Biolabs (catalog VPK-411-DJ). To accomplish muscle-specific expression of transgenes, a HSA (*Acta1*) was PCR amplified from human genomic DNA from a patient’s muscle biopsy. The AAV-HSA-GFP plasmid was developed by inserting the HSA promoter and GFP (ZsGreen1) into a promoterless AAV vector (Cell BioLabs, VPK-411-DJ) using In-Fusion Cloning (Takara Bio, 638911). To generate a CAAHR vector, the mouse AHR coding sequence was PCR amplified from genomic DNA obtained from a C57BL/6J mouse such that the ligand binding domain (amino acids 277–418) was deleted for the murine AHR and subsequently cloned and inserted downstream of the HSA promoter using In-Fusion cloning. The resulting plasmids were packaged using AAV2/9 serotype by Vector Biolabs. The skeletal muscle–specific AAV9s were delivered via several small-volume intramuscular injections of the hindlimb muscle TA, EDL, and gastrocnemius plantar flexor complex at a dosage of 5 × 10^11^ vg/limb. To knock down the AHR in skeletal muscle, we utilized an siRNA sequence (AHR siRNA: 5′-AAG UCG GUC UCU AUG CCG CTT-3′) and a GFP control that were packaged using a mutated AAV9 capsid variant that enables muscle-specific expression (MyoAAV4a) (59) by Vector Biolabs. MyoAAVs were delivered via a tail injection at a dosage of 1 × 10^11^ vg/kg. To generate a transcriptionally deficient CAAHR mutant, we performed Q5 site-directed mutagenesis (New England Biolabs, E0554S) to generate the mutant R39D (61).

### RNA isolation and qPCR.

Total RNA was isolated using TRIzol (Invitrogen, 15-596-018). All samples were homogenized using a PowerLyzer 24 (Qiagen), and RNA was isolated using Direct-zol RNA MiniPrep kit (Zymo Research, R2052). cDNA was generated from 500 ng of RNA using the LunaScript RT Supermix kit (New England Biolabs, E3010L). Real-time PCR (RT-PCR) was performed on a Quantstudio 3 (Thermo Fisher Scientific) using either Luna Universal qPCR Master Mix for Sybr Green primers (New England Biolabs, M3003X) or TaqMan Fast Advanced Master Mix (Thermo Fisher Scientific, 4444557). All primers and TaqMan probes used in this work are listed in Supplemental Table 2. Relative gene expression was calculated as 2^–ΔΔCT^ from the control group.

### Muscle cell culture experiments.

C_2_C_12_ murine myoblasts were obtained from ATCC (catalog CRL-1772) and grown in DMEM (Thermo Fisher Scientific, 10569) supplemented with 10% FBS (VWR, 97068) and 1% penicillin streptomycin (Thermo Fisher Scientific, 15140) at 37°C and 5% CO_2_. All cell culture experiments were performed with low-passage cells (passages 1–5) and in at least 3 biologically independent lots of myoblasts. When assessing AHR activation in muscle cells, C_2_C_12_ myoblasts were incubated for 6 hours with 100 μM of AHR agonist IS, L-Kyn, KA, and IAA. Myoblasts were washed with PBS and collected in TRIzol reagent for total RNA isolation.

### Western blotting.

C_2_C_12_ muscle cells and snap frozen mouse tissue were homogenized in CelLytic M lysis buffer (MilliporeSigma, C2978) supplemented with protease and phosphatase inhibitors (Thermo Fisher Scientific, A32961) in glass Teflon homogenizers and centrifuged at 10,000*g* for 10 minutes at 4°C. The supernatant was collected, and protein quantification was performed using a bicinchoninic acid protein assay (Thermo Fisher Scientific, SL256970). The 2× Laemmli buffer (Bio-Rad, 161-0737) and β-mercaptoethanol (ACROS, 60-24-2) were added to the samples, which were incubated in boiling water for 5 minutes. In total, 10 μL of a prestained ladder (Bio-Rad, 1610394) was loaded in the first lane of a 7.5% Criterion TGX Stain-Free Protein Gel (Bio-Rad, 5678023), while 20 μg (cell lysate) and 100 μg (tissue lysate) of each sample were loaded. Gel electrophoresis was run at 100 V for 1.5 hours and then imaged for total protein on a Bio-Rad imager (GelDoc EZ Imager), before transferring to a polyvinylidene fluoride (PVDF) membrane using a Bio-Rad Trans Blot Turbo system. The PVDF membrane was then imaged for total protein and incubated in blocking buffer (Licor, 927-60001) for 1 hour at room temperature while rocking. The membrane was incubated overnight at 4°C with AHR primary antibody (NSJ Bioreagents, R30877, 1 μg/mL), PhosphoDetect anti–PDH-E1α (pSer^300^) primary antibody (MilliporeSigma, AP1064, 0.2 μg/mL), or PDK4 primary antibody (ProteinTech, 12949-1-AP, 1:1,000) in blocking buffer. After overnight incubation, the membranes were washed 3× 10 minutes with TBS + 0.01% Tween. The membranes were then incubated for 2 hours in blocking solution with secondary antibody (Licor, C80118-05, 1:10,000 dilution) to detect the AHR, PDH-E1α (pSer^300^), and PDK4, while the total PDHE1α antibody was conjugated to Alexa Fluor 790 (Santa Cruz Biotechnology Inc., 377092AF790). Next, the membranes were then washed 3× 10 minutes in TBS + 0.01% Tween and imaged on a Licor Odyssey CLx. Uncropped blots and gel images are provided in Supplemental Figures 1 and 9.

### RNA validation of skeletal muscle–specific KO of the AHR.

The soleus muscle was dissected from healthy AHR^fl/fl^ mice and AHR^mKO^ mice and incubated in Krebs buffer supplemented with 10 mM glucose and gassed with 95% O_2_ and 5% CO_2_ at 37°C. The muscles were treated with 500 μM IS or equal volume of DMSO for 3.5 hours and then processed for qPCR analysis.

### Induction of CKD.

Two weeks after tamoxifen treatment, mice were assigned to a casein-based chow diet for 7 days, followed by induction of CKD via the addition of 0.2% (w/w) adenine to the diet. CKD mice were kept on 0.2% adenine diet for the duration of the study. Control mice were fed a casein-based chow diet for the entirety of the experiment.

### Delivery of probenecid.

Mice were administered i.p. injections of 25 mg/kg of probenecid twice daily (Invitrogen, P36400) or PBS (vehicle control) starting 2 weeks after CKD induction, for the duration of 2 weeks. On the last day of injections, probenecid or PBS was administered 2 hours prior to euthanasia. Plasma was isolated and stored at –80°C for further metabolomic analyses described below.

### Targeted metabolomics in mouse plasma.

Targeted metabolomic analyses were performed by the Southeast Center for Integrated Metabolomics at the University of Florida. Under ketamine (100 mg/kg) and xylazine (10 mg/kg) anesthesia, blood was collected via cardiac puncture using a heparin-coated syringe, centrifuged at 1,200*g* at 4°C for 10 minutes, and plasma was stored at –80°C until analysis. Plasma was processed as done previously (22, 24).

### Assessment of renal function.

GFR was evaluated by measuring FITC-labeled inulin clearance (100, 101). GFR was assessed via blood collection from a 1 mm tail snip at multiple time points (3, 5, 7, 10, 15, 35, 56, 75 minutes) following retro-orbital injection of FITC-labeled inulin (MilliporeSigma, F3272) in heparin-coated capillary tubes. Blood collected was centrifuged at 1,200*g* at 4°C for 10 minutes, and plasma was diluted (1:20) and loaded into a 96-well plate along with a FITC-inulin standard curve; fluorescence was detected using a BioTek Synergy II plate reader. GFR was calculated using a 2-phase exponential decay. BUN was assessed from plasma collected prior to euthanasia using a commercial kit (Arbor Assays, K024).

### Assessment of forelimb grip strength.

Bilateral forelimb grip strength was assessed using a grip strength meter (BIOSEB, BIO-GS3). Mice were encouraged to firmly grip the metal T-bar and were pulled backward horizontally with increasing force until they released the T-bar. Three trials were performed, allowing the mice 30 seconds to rest between each trial, and the highest force was analyzed.

### Peroneal nerve–stimulated EDL force frequency and fatigue analysis.

Mice were anesthetized with an i.p. injection of xylazine (10 mg/kg) and ketamine (100 mg/kg), and the distal portion of the extensor digitorum longus (EDL) tendon was sutured with a double square knot using 4–0 silk suture, and the tendon was carefully cut distal to the suture. The mouse was placed prone on a thermoregulated platform (37°C), and the knee was immobilized/stabilized with a pin attached to the platform. The suture attached to the distal end of the EDL tendon was secured to a force length transducer (Cambridge Technology, model 2250), and 2 Chalgren electrodes (catalog 111-725-24TP) were placed on both sides of the peroneal nerve and connected to an Aurora Scientific stimulator (701A stimulator). Data were collected using the DMC program (version v5.500, Aurora Scientific). Optimal length was determined by recording force production of twitch contractions while incrementally increasing muscle length with 60 seconds of rest between each contraction. Once optimal length was achieved, the EDL underwent a force frequency assessment by stimulating the peroneal nerve at 1, 25, 50, 75, 100, 125, 150, and 175 Hz (spaced 1 minute apart) using 2.4 mAmp stimulation, 0.1 ms pulse width, and a train duration of 0.5 seconds. Specific force was calculated by normalizing absolute force production to the EDL mass. Following force frequency analysis, the EDL was rested for 2 minutes before undergoing a series of 80 contractions at 50 Hz (2.4 mAmp stimulation, 0.1 ms pulse width, and train duration of 0.5 seconds) performed every 2 seconds to assess fatiguability of the muscle.

### Mitochondrial isolation.

Skeletal muscle mitochondria were isolated from the gastrocnemius and quadriceps muscles. Dissected muscles were immediately placed in ice-cold Buffer A (phosphate buffered saline supplemented with EDTA [10 mM], pH 7.4) and trimmed to remove connective tissue and fat before it was minced and subjected to a 5-minute incubation on ice in Buffer A supplemented with 0.025% trypsin (MilliporeSigma, T4799). Following trypsin digestion, skeletal muscle was centrifuged at 500*g* at 4°C for 5 minutes, and the supernatant was aspirated to remove trypsin. Digested muscle tissue was resuspended in Buffer C — MOPS (50 mM), KCl (100 mM), EGTA (1 mM), MgSO_4_ (5 mM), BSA (2 g/L); pH 7.1 — and homogenized via a glass-Teflon homogenizer (Wheaton) for approximately 5 passes before being centrifuged at 800*g* at 4°C for 10 minutes. The resulting supernatant was collected in a separate tube and centrifuged at 10,000*g* at 4°C for 10 minutes to pellet mitochondria. All steps were performed at 4°C. The mitochondrial pellet was gently washed with Buffer B — MOPS (50 mM), KCl (100 mM), EGTA (1 mM), MgSO_4_ (5 mM); pH 7.1 — to remove damaged mitochondria on the exterior of the pellet and then resuspended in Buffer B. Protein concentration was determined using a bicinchoninic acid protein assay (Thermo Fisher Scientific, A53225).

### Skeletal muscle mitochondrial OXPHOS function.

High-resolution respirometry was measured using Oroboros Oxygraph-2k (O2K) measuring *J*O_2_ at 37°C in Buffer D (105 mM K-MES, 30 mM KCl, 1 mM EGTA, 10 mM K_2_HPO_4_, 5 mM MgCl_2_-6H_2_O, 2.5 mg/mL BSA, pH 7.2) supplemented with 5 mM creatine (Cr). A CK clamp was employed to leverage the enzymatic activity of CK, which couples the interconversion of ATP and ADP to that of PCr and free Cr, to titrate the extra mitochondrial ATP/ADP ratio; thus, the free energy of ATP hydrolysis (ΔG_ATP_) could be calculated (102). This approach allows assessment of mitochondrial flux across a range of physiological relevant energetic demands (ΔG_ATP_, heavy exercise to rest), which are controlled by altering the PCr/Cr ratio. The ΔG_ATP_ can be plotted against the corresponding *J*O_2_ creating a linear force-flow relationship, where the slope represents the conductance through the OXPHOS system. In total, 25 μg of mitochondria were added to the Oxygraph chamber in 2 mL of Buffer D supplemented with ATP (5 mM), Cr (5 mM), PCr (1 mM), and CK (20 U/mL) at 37°C. Conductance measurements were performed using various combinations the following substrates: pyruvate (5 mM), malate (2.5 mM), and octanoyl-L-carnitine (0.2 mM). In all experiments, exogenous cytochrome *c* was added to confirm the outer mitochondrial membrane was intact. A list of key chemicals, including suppliers and catalog numbers, can be found in Supplemental Table 3.

### JNAD(P)H matrix dehydrogenase assays.

Matrix dehydrogenase function was assessed utilizing the autofluorescence of NADH or NADPH (Ex/Em = 340/450) in a 96-well plate using a kinetic protocol on a BioTek Synergy 2 Multimode Microplate Reader. For all assays, Buffer D was supplemented with alamethicin (0.03 mg/mL), rotenone (0.005 mM), NAD^+^ (2 mM), or NADP^+^ (2 mM). Dehydrogenase enzymes such as PDH and AKGDH required supplementation of cofactors Coenzyme A (0.1 mM) and thiamine pyrophosphate (TPP; 0.3 mM). Prewarmed Buffer D (37°C) was loaded in a 96-well plate followed by the addition of mitochondria. Dehydrogenase activity was initiated with the addition of enzyme-specific fuel sources: pyruvate (5 mM, PDH), glutamate (10 mM, GDH), malate (5 mM, malate dehydrogenase [MDH] and ME), α-ketoglutarate (10 mM, AKGDH), citrate (6 mM, aconitase), fumarate (10 mM, FH), hydroxybutyrate (10 mM, β hydroxy butyrate dehydrogenase [βHBDH]), or isocitrate (5 mM, isocitrate dehydrogenase 2 and 3 [ICDH2/3]). Rates of NADH/NADPH production was calculated as a slope of linear portions of NADH/NADPH curves and converted to pmols of NADH/NADPH by a standard curve.

### Complex V activity (ATP synthase).

Mitochondria were lysed in Cell Lytic M, and enzyme activity was measured in Buffer E (2.5 mM MgCl_2_-6H_2_O, 20 mM HEPES, 100 mM KCl, 2.5 mM KH_2_PO_4_, 1% glycerol, pH 8.0) supplemented with lactate dehydrogenase (10 mM), pyruvate kinase (10 mM), rotenone (0.005 mM), phospho-enol-pyruvate (PEP, 5 mM), and NADH (0.2 mM). In this assay, the ATP synthase works in reverse (hydrolysis of ATP), as the mitochondrial membrane potential was dissipated by lysis. Using a pyruvate kinase/lactate dehydrogenase–coupled assay, ATP hydrolysis (by the ATP synthase) is coupled to NADH consumption in a 1:1 stoichiometry. The rate of decay of NADH autofluorescence (Ex/Em = 340/450 nm) represents ATP synthase activity. Fluorescence values were converted to pmols of NADH by a standard curve.

### Immunofluorescence microscopy.

Transverse sections (10 μm–thick) were cut from the tibialis anterior, EDL, and soleus muscles mounted in optimal cutting temperature compound and frozen in liquid nitrogen–cooled isopentane using a Leica 3050S cryotome. Muscle sections were fixed with 4% paraformaldehyde in PBS for 5 minutes at room temperature, followed by 10 minutes of permeabilization using 0.25% (v/v) Triton X-100 in PBS. Next, sections were washed with PBS 3 times for 2 minutes each wash. Sections were blocked for 1 hour at room temperature with blocking buffer (PBS supplemented with 5% goat serum and 1% BSA). Sections were incubated overnight at 4°C with a primary antibody against laminin (1:100 dilution, MilliporeSigma, L9393) to label myofiber membranes. Following 4 PBS washes, sections were incubated for 1 hour with Alexa Fluor secondary antibodies (Thermo Fisher Scientific, 1:100 dilution), then were washed again 4 times using PBS (5 minutes each); coverslips were mounted with Vectashield hardmount containing DAPI (Vector Laboratories, H1500) to label nuclei. Muscle sections were imaged at ×20 magnification using an Evos FL2 Auto microscope. All images were analyzed for CSA using MuscleJ (103).

### ATAC-Seq.

Nuclei were isolated by gentle homogenization (10 mM Tris-HCl [pH 7.5], 10 mM NaCl, 3 mM MgCl_2_, 0.1% Tween-20, 0.1% NP-40, and 0.01% Digitonin) of skeletal muscle followed by tagmentation (Tagment DNA buffer and Tn5, Illumina) for 30 minutes at 37°C. DNA was then purified with the MinElute purification kit from Qiagen. The purified DNA was PCR amplified for 15 cycles using Q5 High Fidelity DNA polymerase (New England Biolabs, M0491S) with the incorporation of Illumina Nextera XT adaptors (Illumina). The libraries were then size selected with AmpureXP Beads (Beckman, A63880). Quality control of the libraries was verified using a bioanalyzer. Libraries were sequenced on Illumina HiSeq4000 using paired end (PE) 150 bp. The reads were first mapped to the GRCm39-mm39 mouse genome assembly using Bowtie2 version 2.1.0. Mitochondrial, duplicate, and nonunique reads were removed before peak calling. MACS2 was used for peak calling employing BAMPE mode. Differentially expressed peaks were identified using edgeR.

### Statistics.

Data are presented as the mean ± SD. Normality of data was assessed using the Shapiro-Wilk test. Data without normal distribution were analyzed using a Kruskal-Wallis test. Data involving comparisons of 2 groups were analyzed using a 2-tailed Student’s *t* test. Data involving comparisons of more than 2 groups were analyzed using either a 1-way ANOVA with Tukey’s post hoc or a 2-way ANOVA with Dunnett’s post hoc testing for multiple comparisons when significant interactions were detected. Pearson correlations involved 2-tailed statistical testing. All analyses were performed in GraphPad Prism (Version 9.5.1). *P* < 0.05 was considered significant.

### Study approval.

All human experiments in this study were approved by the IRBs (IRB201801553) at the University of Florida and the Malcom Randall VA Medical Center (Gainesville, Florida, USA). All study procedures were carried out according to the Declaration of Helsinki, and participants were fully informed about the research; informed consent was obtained. All animal experiments in this study were approved by the IACUC of the University of Florida (protocol no. 202110484). All animal experiments adhered to the *Guide for the Care and Use of Laboratory Animals* (National Academies Press, 2011).

### Data availability.

Values for all data points in graphs are reported in the Supporting Data Values file. Raw sequencing data have been uploaded to the Gene Expression Omnibus (accession no. GSE255812).

## Author contributions

TT and TER designed the study; TT, NAV, LES, KW, STS, and TER conducted experiments. TT, NAV, LES, KW, and TER analyzed data. TT, NAV, LES, KW, STS, and TER interpreted the data. TT and TER drafted the manuscript. TT, NAV, LES, KW, STS, and TER edited and revised the manuscript. All authors approved the final version of this manuscript.

## Supplementary Material

Supplemental data

Supporting data values

## Version 1 04/23/2024

In-Press Preview

## Version 2 05/22/2024

Electronic publication
