# Supplementary material for: A tryptophan-derived uremic metabolite/Ahr/Pdk4 axis governs skeletal muscle mitochondrial energetics in chronic kidney disease
Source: JCI Insight. 2024 Apr 23;9(10):e178372. doi: 10.1172/jci.insight.178372 (PMC11141944; doi:10.1172/jci.insight.178372)
Supplement: Supplemental data [file jciinsight-9-178372-s215.pdf]

SUPPLEMENTARY MATERIAL

A tryptophan-derived uremic metabolite-AHR-Pdk4 axis governs skeletal muscle mitochondrial energetics in chronic kidney disease

Trace Thome[1], Nicholas A. Vugman[1], Lauren E. Stone[1], Keon Wimberly[1], Salvatore T. Scali[2,5], Terence E. Ryan[1,3,4,#]

[1]Department of Applied Physiology and Kinesiology, [2]Division of Vascular Surgery and Endovascular Therapy, [3]Myology Institute, [4]Center for Exercise Science, University of Florida, Gainesville, FL, USA.

[5]Malcom Randall VA Medical Center, Gainesville, FL, USA.

#Correspondence should be addressed to Terence E. Ryan, PhD: 1864 Stadium Rd, Gainesville, FL, 32611. Tel: 352-294-1700 (office); email: ryant@ufl.edu

Content Included:

- Supplemental Tables 1-3
- Supplemental Figures 1-10

Supplemental Table 1: Physical and Clinical Characteristics of Human Patients

**Characteristic**	**Control** (N=12)	**CKD** (N=10)	**P value** (X^2 or *t*-test)
Age (years) – (SD)	66.2 (6.6)	66.2 (11.5)	0.99
Female sex – no. (%)	4 (33)	4 (40)	0.86
BMI (kg/m^2) – (SD)	29.5 (7.6)	27.7 (7.9)	0.59
eGFR (mL/min/1.73*m^2) – (SD)	85.67 (14.77)	19.30 (10.34)	**1.45E-10**
Creatinine (mg/dL) – (SD)	0.88 (0.24)	4.55 (2.43)	**3.94E-05**
BUN (mg/dL) – (SD)	17.5 (5.9)	33.8 (13.4)	**0.0011**
Albumin (g/dL) – (SD)	4.23 (0.39)	4.53 (1.1)	0.48
Medical history – no. (%)			
Diabetes mellitus type I or II	7 (58)	3 (30)	0.36
Hypertension	8 (67)	10 (100)	0.14
Hyperlipidemia	6 (50)	7 (70)	0.61
Coronary artery disease	6 (50)	4 (40)	0.99
Congestive Heart Failure	4 (33)	3 (30)	0.76
Medication used – no. (%)			
Aspirin	7 (58)	5 (50)	0.99
Statin	11 (92)	7 (70)	0.45
ACE inhibitor	3 (25)	3 (30)	0.82
Anticoagulant	1 (8)	2 (20)	0.86
Antiplatelet	3 (25)	3 (30)	0.82

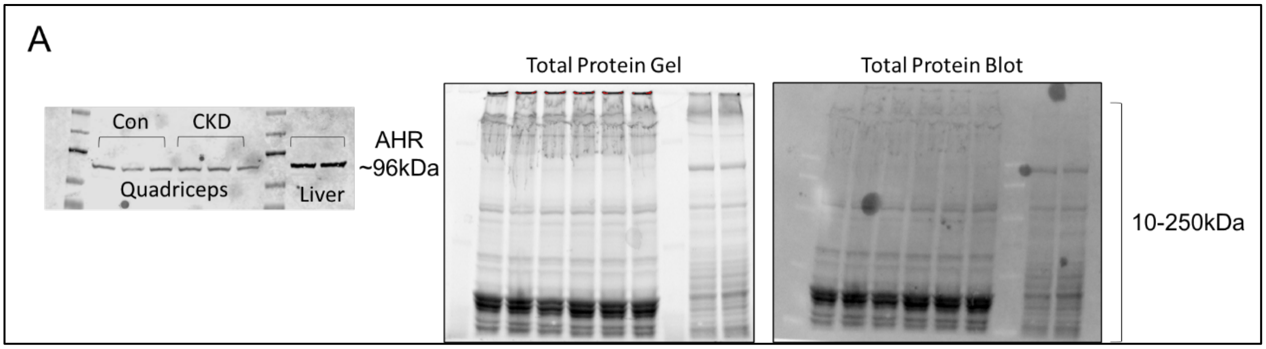

Supplemental Figure 1. (A) Uncropped gel and PVDF membranes for western blot analysis confirming AHR protein expression is present in skeletal muscle (quadriceps) of control and CKD mice.

Supplemental Table 2.

Primers and Probes for RT-PCR analysis			
**Gene Name**	**Chemistry**	**Sequence or Product Number**	**Species**
Actb	Sybr Green	5’-GGCTGTATTCCCCTCCATCG-3’	Mus Musculus
		5’-CCAGTTGGTAACAATGCCATGT-3’	
Ahrr	Sybr Green	5’- GACTTCTGCAGACAGCTACA-3’	Mus Musculus
		5’-TGTCAAGAAGGCCGAGTACT-3’	
Ahr Exon 1-2	Sybr Green	5’-AACATCACCTATGCCAGCCG-3’	Mus Musculus
		5’-GGTCTCTGTGTCGCTTAGAAGG-3’	
Ahr Exons 2-3	Sybr Green	5’-AAGCTGGACAAACTCTCTGTTCTT-3’	Mus Musculus
		5’-GCCAGTCTCTGATTTGTGCTCTA-3’	
Arnt	Sybr Green	5’-CATCATCGGTCTAGTTCCAGTG-3’	Mus Musculus
		5’-CAGCATGGACAGCATTTCTTG-3’	
Atp5d	Sybr Green	5’-TGCTTCAGGCGCGTACATAC-3’	Mus Musculus
		5’-CACTTGCTTGACGTTGGCA-3’	
Atp5k	Sybr Green	GTTCAGGTCTCTCCACTCATCA	Mus Musculus
		CGGGGTTTTAGGTAACTGTAGC	
Cox7a1	Sybr Green	GCTCTGGTCCGGTCTTTTAGC	Mus Musculus
		GTACTGGGAGGTCATTGTCGG	
Cox8b	Sybr Green	GCGAAGTTCACAGTGGTTCC	Mus Musculus
		GGAACCATGAAGCCAACGAC	
Cs	Sybr Green	GGACAATTTTCCAACCAATCTGC	Mus Musculus
		TCGGTTCATTCCCTCTGCATA	
Cyp1a1	Sybr Green	CAGCCTTCCCAAATGGTTTA	Mus Musculus
		GCCTGGGCTACACAAGACTC	
Cyp1b1	Sybr Green	AGGATGTGCCTGCCACTATT	Mus Musculus
		AGCTGGAGAATCGCATTGAT	
Errα_1	Sybr Green	GGAGGACGGCAGAAGTACAAA	Mus Musculus
		GCGACACCAGAGCGTTCAC	
L32	Sybr Green	TTCCTGGTCCACAATGTCAA	Mus Musculus
		GGCTTTTCGGTTCTTAGAGGA	
Ndufa5	Sybr Green	AGCTGGATATGGTCAAGGCG	Mus Musculus
		GCCACTTCCACTGGTTAGCA	
Pdha1	Sybr Green	GAAATGTGACCTTCATCGGCT	Mus Musculus
		TGATCCGCCTTTAGCTCCATC	
Pdk1	Sybr Green	GGCCAGGTGGACTTCTATGC	Mus Musculus
		AGCATTCACTGACCCGAAGT	
Pdk2	Sybr Green	GGCGCTGTTGAAGAATGCG	Mus Musculus
		GGCATTGCTGGATCCGAAGTC	
Pdk3	Sybr Green	GCCCGGTACTTGTGTAGGTG	Mus Musculus
		TTACCTCTACCTGGGGCTCG	
Pdk4	Sybr Green	CCGCTGTCCATGAAGCA	Mus Musculus
		GCAGAAAAGCAAAGGACGTT	
Pdp1	Sybr Green	CGGCTCCGTGTTGTGATGA	Mus Musculus
		TCTGACTGGGATTCCAATTCGT	

Pdp2	Sybr Green	GGACGAGGATACGAGGCTGA	Mus Musculus
		GCGTCTCCCACCTCGTAAAA	
Sdhb	Sybr Green	AATTTGCCATTTACCGATGGGA	Mus Musculus
		AGCATCCAACACCATAGGTCC	
Sod2	Sybr Green	ACAAACCTGAGCCCTAAGGGT	Mus Musculus
		GAACCTTGGACTCCCACAGAC	
Tbp	Sybr Green	ATCCCAAGCGATTTGCTG	Mus Musculus
		CCTGTGCACACCATTTTTCC	
Tfam	Sybr Green	ATTCCGAAGTGTTTTTCCAGCA	Mus Musculus
		TCTGAAAGTTTTGCATCTGGGT	
Ahr	Taqman	Mm00478932	Mus Musculus
AHR	Taqman	Hs00169233	Homo Sapiens
Cyp1a1	Taqman	Mm00487218	Mus Musculus
CYP1A1	Taqman	Hs00153120	Homo Sapiens
Cyp1b1	Taqman	Mm00487229	Mus Musculus
CYP1B1	Taqman	Hs00164383	Homo Sapiens
**Primers used for Validation of DNA Recombination in AHR^{mKO} Mice**			
**Gene Name**	**Primer**	**Sequence**	**Species**
Ahr	Forward	5’-ATCTTGTGTCAGGAACAGGCCATC-3’	Mus Musculus
	Reverse	5’-GGTACAAGTGCACATGCCTGC-3’	

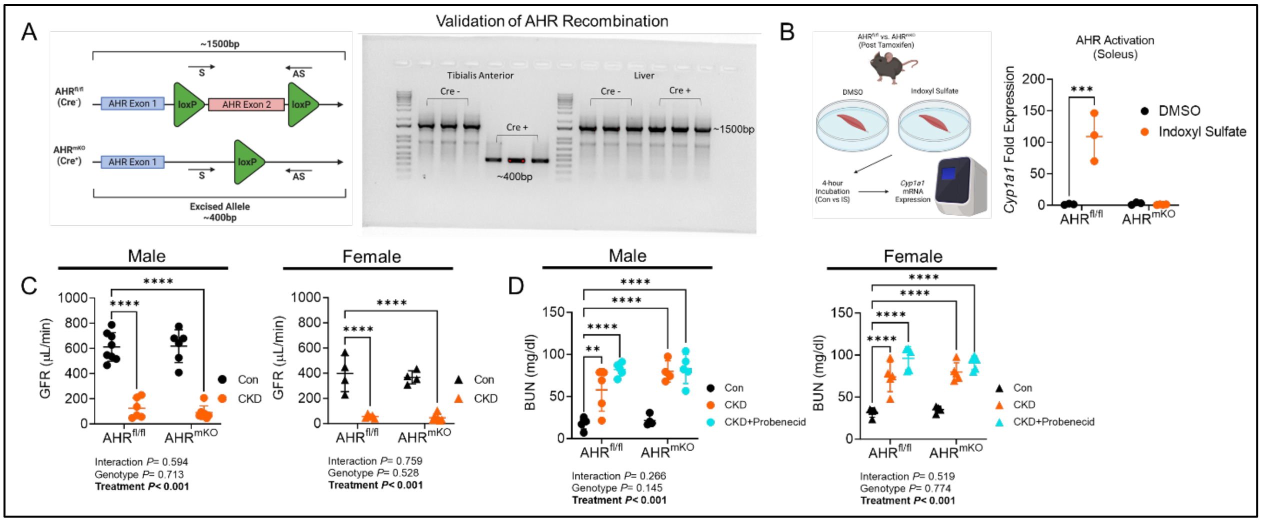

Supplemental Figure 2. (A) Graphical depiction of Cre-mediated excision of exon 2 and the DNA gel confirming muscle-specific recombination in the AHR gene in AHR^{mKO} mice treated with tamoxifen. (B) Graphical illustration of experimental procedure to validate AHR dependent mRNA signaling was abolished in AHR^{mKO} mice and quantification of IS induced *Cyp1a1* gene expression (n=3/genotype/group). (C) Validation of reduced glomerular filtration rate (GFR, n=4-8/group/sex) and (D) elevated plasma blood urea nitrogen (BUN, n=3-5/group/sex) concentrations. Statistical analyses performed using two-way ANOVA with Dunnett's post hoc testing for multiple comparisons when significant interactions were detected. Error bars represent the standard deviation. $^{**}P<0.01$, $^{***}P<0.001$, $^{****}P<0.0001$.

Supplemental Table 3.

Chemical Name	Supplier	Catalog No.
α-Ketoglutaric acid	Millipore-Sigma	K1750
α-keto-β-Methylvalerate	Millipore-Sigma	198978
Adenosine 5’ -diphosphate (ADP)	Millipore-Sigma	A5285
Adenosine 5’-triphosphate di(tris) salt hydrate (ATP)	Millipore-Sigma	A9062
Alamethecin from *Trichoderma viride*	Millipore-Sigma	A4665
Amplex Ultra Red (AUR)	Millipore-Sigma	A36006
Antimycin A (AMA)	Millipore-Sigma	A8674
Auranofin	Millipore-Sigma	A6733
β-Nicotinamide adenine dinucleotide hydrate (NADH)	Millipore-Sigma	N1636
β-Nicotinamide adenine dinucleotide salt (NAD+)	Millipore-Sigma	N0632
(NADPH)	Millipore-Sigma	N1630
(NADP+)	Millipore-Sigma	N5755
Bovine serum albumin (BSA)	Millipore-Sigma	A7030
Cell lytic M	Millipore-Sigma	C2978
Creatine Kinase (CK)	Millipore-Sigma	C3755
Creatine monohydrate	Millipore-Sigma	C3630
Coenzyme A trilithium salt	Millipore-Sigma	C3019
Cytochrome c from equine heart	Millipore-Sigma	C2506
D-Glucose	Millipore-Sigma	G32030
EDTA	Millipore-Sigma	E9884
EGTA	Millipore-Sigma	E3889
Fetal Bovine Serum (FBS)	Avantar (VWR)	97068
Goat Serum	ThermoFisher Scientific	16210064
Hydrogen Peroxide, 30%	ThermoFisher Scientific	H325-100
Horseradish Peroxidase (HRP)	Millipore-Sigma	P8375
Insulin/transferrin/selenium	ThermoScientific	41400
Indoxyl sulfate potassium salt	Millipore-Sigma	I3875
Indole-3-acetic acid (IAA)	Millipore-Sigma	I5148
Isocitrate	Millipore-Sigma	58790
Isocitrate dehydrogenase (ICDH)	Millipore-Sigma	I1877
Kynurenic Acid (KA)	Millipore-Sigma	K3375
L-Kynurenine (L-Kyn)	Millipore-Sigma	K8625
Lactate dehydrogenase/pyruvate kinase	Millipore-Sigma	P0294
Magnesium chloride hexahydrate	Research Products International	M24000
Magnesium Sulfate	Millipore-Sigma	M2643
Malate Dehydrogenase	Millipore-Sigma	442610

Malic acid	Millipore-Sigma	M7397
Malonate	Millipore-Sigma	M1296
MES potassium salt	Millipore-Sigma	M0895
2-Methylbutane	Fisher Scientific	60048070
MOPS	Millipore-Sigma	M1254
Octanoyl-L-carnitine (OC)	Millipore-Sigma	50892
Oligomycin A	Millipore-Sigma	75351
Penicilin-streptomycin	ThermoFisher Scientific	15140
Pierce rapid gold BCA protein assay kit	ThermoFisher Scientific	A53225
Phosphoenol-pyruvate (PEP)	Millipore-Sigma	10108294001
Potassium chloride (KCl)	Millipore-Sigma	P9541
Potassium phosphate (K_2HPO_4) dibasic	Millipore-Sigma	P3786
Potassium Phosphate (KH_2PO_4) monobasic	Millipore-Sigma	P2670
Potassium Pyruvate	Combi-Blocks	QA1116
Rotenone	Millipore-Sigma	R8875
Succinic acid	Research Products International	S42000
Superoxide dismutase from bovine erythrocytes	Millipore-Sigma	S7446
Thiamine pyrophosphate (TPP)	Millipore-Sigma	C8754
Tris-adenosine trisphosphate (ATP)	Millipore-Sigma	A9062
Tris-phosphocreatine (PCr)	Millipore-Sigma	P1937
Triton-X100	Millipore-Sigma	X100
Trypsin	Millipore-Sigma	T4799
Trypsin-EDTA (0.25%)	ThermoFisher Scientific	252000

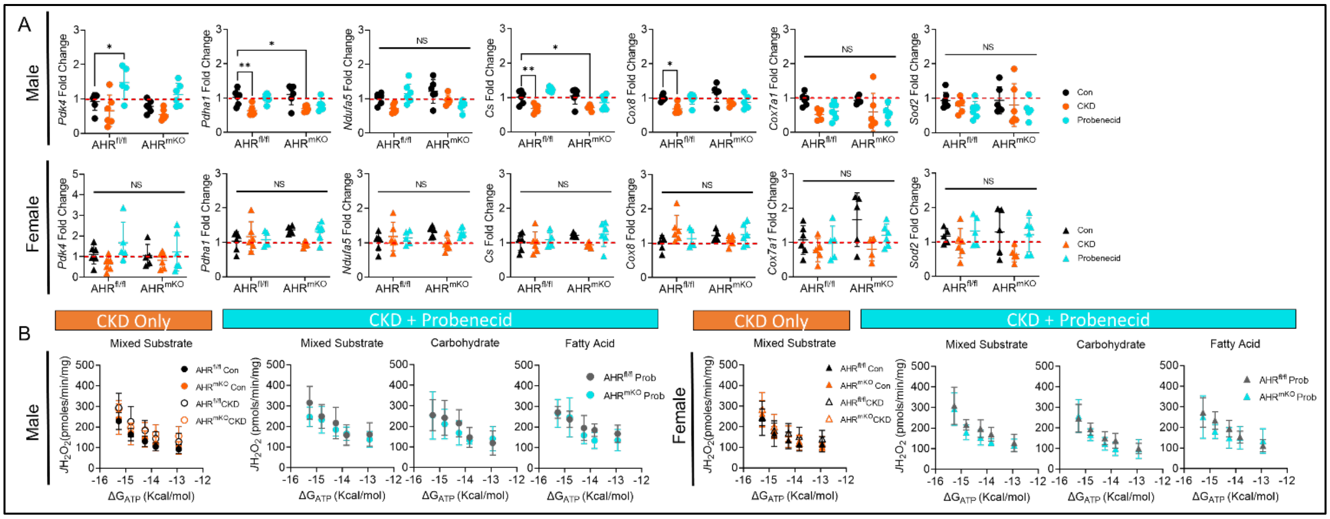

Supplemental Figure 3. (A) qPCR analysis of genes related to mitochondrial health and function in AHR$^{fl/fl}$ and AHR^{mKO} male/female control mice, CKD only mice, and CKD mice administered probenecid daily (n=4-7/group). (B) Relationship between JH$_2$O$_2$ and ΔG_{ATP} for male and female AHR$^{fl/fl}$ and AHR^{mKO} with CKD and administered probenecid daily when mitochondria were energized with carbohydrates only (pyruvate/malate), fatty acids only (octanoyl-L-carnitine/malate), or mixed carbohydrate and fatty acid substrates (pyruvate/malate/octanoyl-L-carntine) (n=4-9/group). Statistical analyses performed using two-way ANOVA with Dunnett's post hoc testing for multiple comparisons when significant interactions were detected. Error bars represent standard deviation.

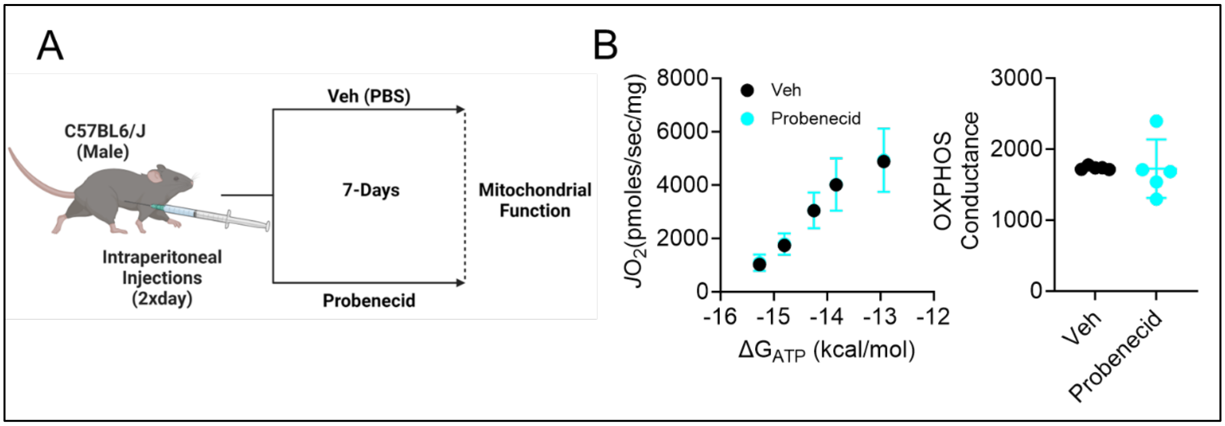

Supplemental Figure 4. (A) Graphical depiction of the experimental approach testing if daily systemic injections of probenecid negatively impact mitochondrial OXPHOS conductance in healthy control mice. (B) Relationship between JO_2 and ΔG_{ATP} in isolated mitochondria from the gastrocnemius muscle using carbohydrate (pyruvate/malate) fuel sources in male mice treated with vehicle (Veh, PBS) or probenecid (25mg/kg), and quantification of OXPHOS conductance (n=5/group). Statistical analyses were performed using two-tailed Student's *t*-test. Error bars represent standard deviation.

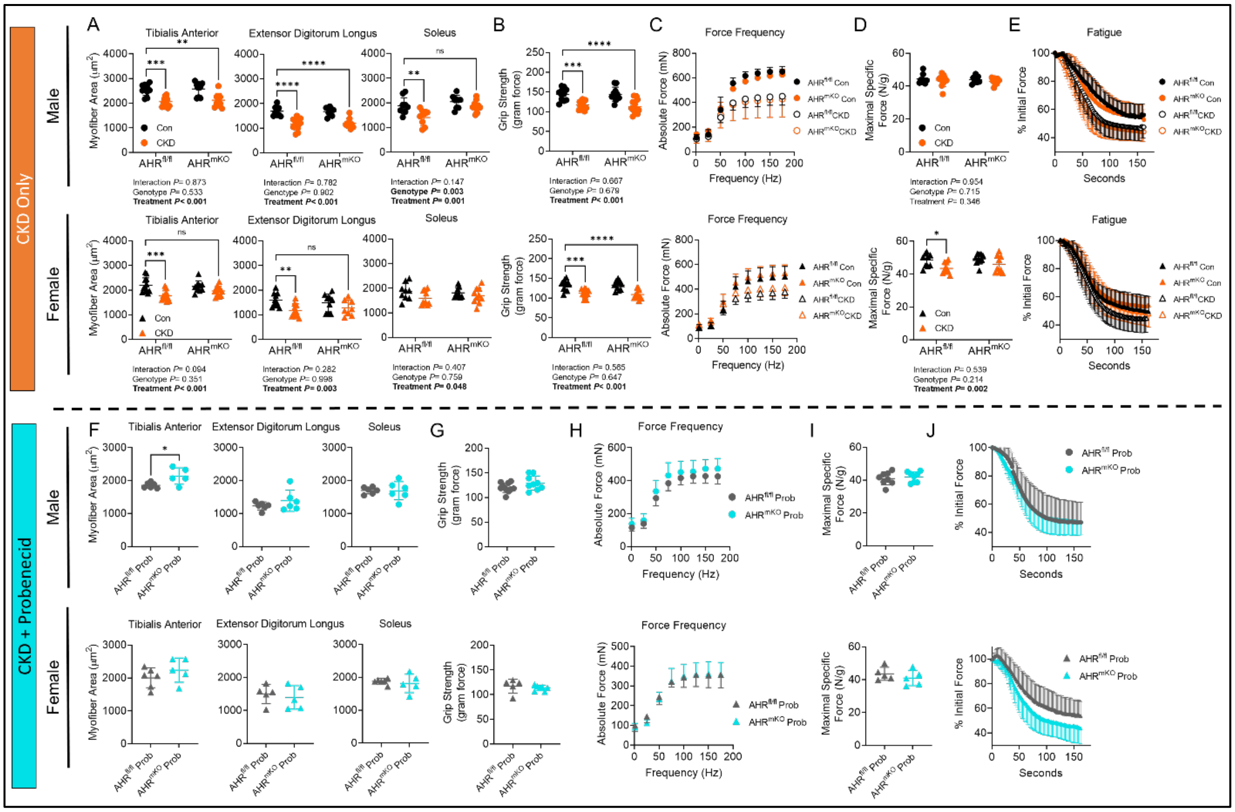

Supplemental Figure 5. (A) Myofiber area of the tibialis anterior, extensor digitorum longus, and soleus of male and female AHR$^{fl/fl}$ and AHR^{mKO} mice with CKD. (B) *In vivo* dual forelimb grip strength, (C) *in situ* peroneal nerve stimulated force frequency analysis and (D) maximal specific force production quantification, and (E) muscle fatigue analysis of the extensor digitorum longus muscle in male AHR$^{fl/fl}$ and AHR^{mKO} mice with CKD (n=7-10/group). (F) Myofiber area measurements of the tibialis anterior, extensor digitorum longus, and soleus of male and female AHR$^{fl/fl}$/AHR^{mKO} mice treated with CKD and probenecid. (G) Comparisons of *in vivo* dual forelimb grip strength. (H) *In situ* force frequency, (I) maximal specific force quantification, and (J) fatigue analysis of the extensor digitorum longus in AHR$^{fl/fl}$/AHR^{mKO} mice with CKD and probenecid. Statistical analyses performed using Student's *t*-tests or two-way ANOVA with Dunnett's post hoc testing for multiple comparisons when interactions were detected. Error bars represent standard deviation. **P*<0.05, ***P*<0.01, ****P*<0.001, *****P*<0.0001. NS = no significance.

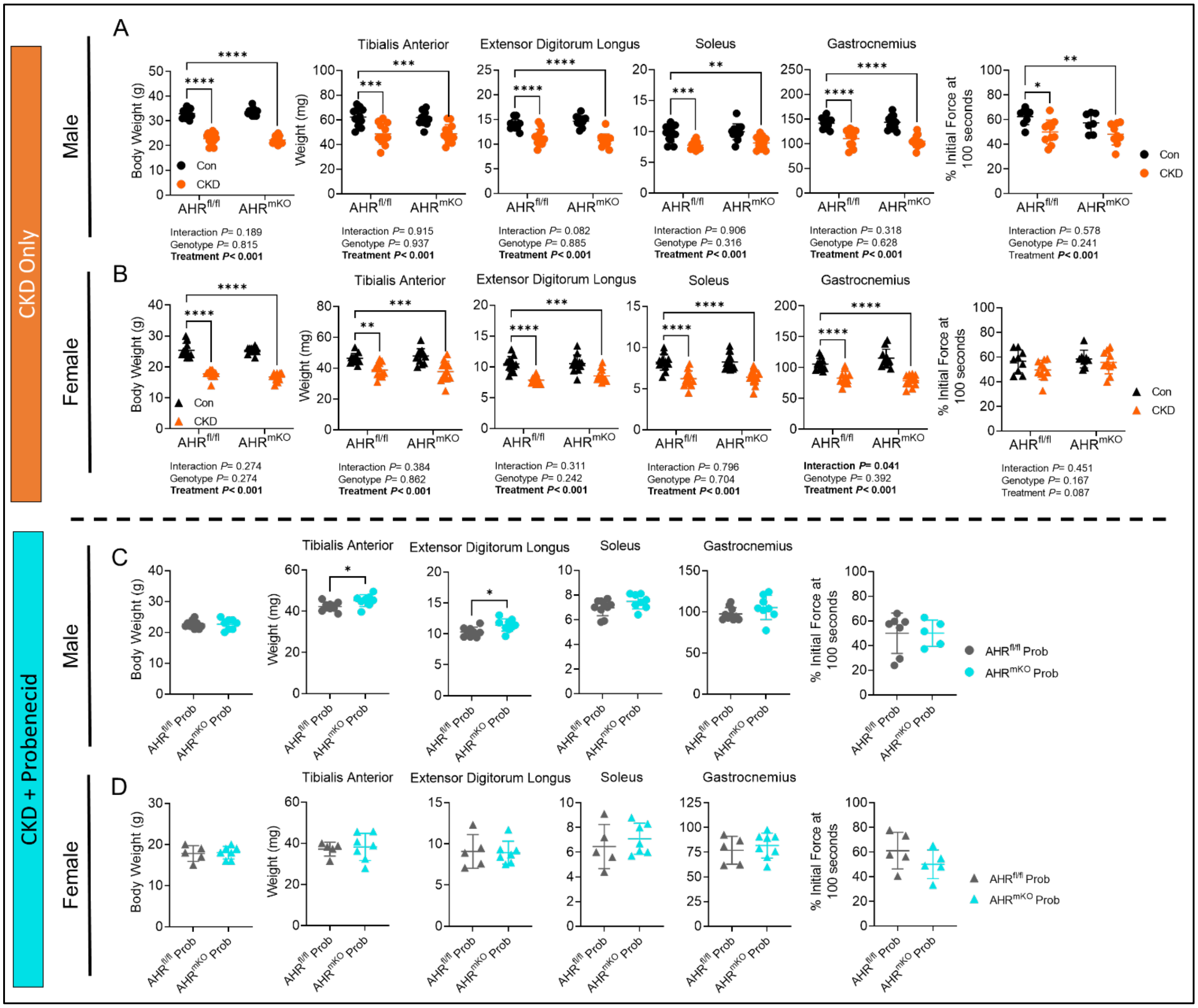

Supplemental Figure 6. (A) Body weights, muscle wet weights (tibialis anterior, extensor digitorum longus, soleus, and gastrocnemius), and extensor digitorum longus muscle fatigue quantification in male $AHR^{fl/fl}$ and AHR^{mKO} mice with CKD only (n=7-12/group). (B) Identical measurements in female $AHR^{fl/fl}$ and AHR^{mKO} mice with CKD only (n=8-12/group). (C) Quantification of body weights, muscle wet weights (tibialis anterior, extensor digitorum longus, soleus and gastrocnemius), and extensor digitorum longus muscle fatigue in male $AHR^{fl/fl}$ and AHR^{mKO} mice with CKD and administered probenecid daily (n=5-10/group). (D) Identical measurements in female $AHR^{fl/fl}$ and AHR^{mKO} mice with CKD only (n=5-10/group). Statistical analyses performed using Student's *t*-tests or two-way ANOVA with Dunnett's post hoc testing for multiple comparisons when interactions were detected. Error bars represent standard deviation. *P<0.05, **P<0.01, ***P<0.001, ****P<0.0001.

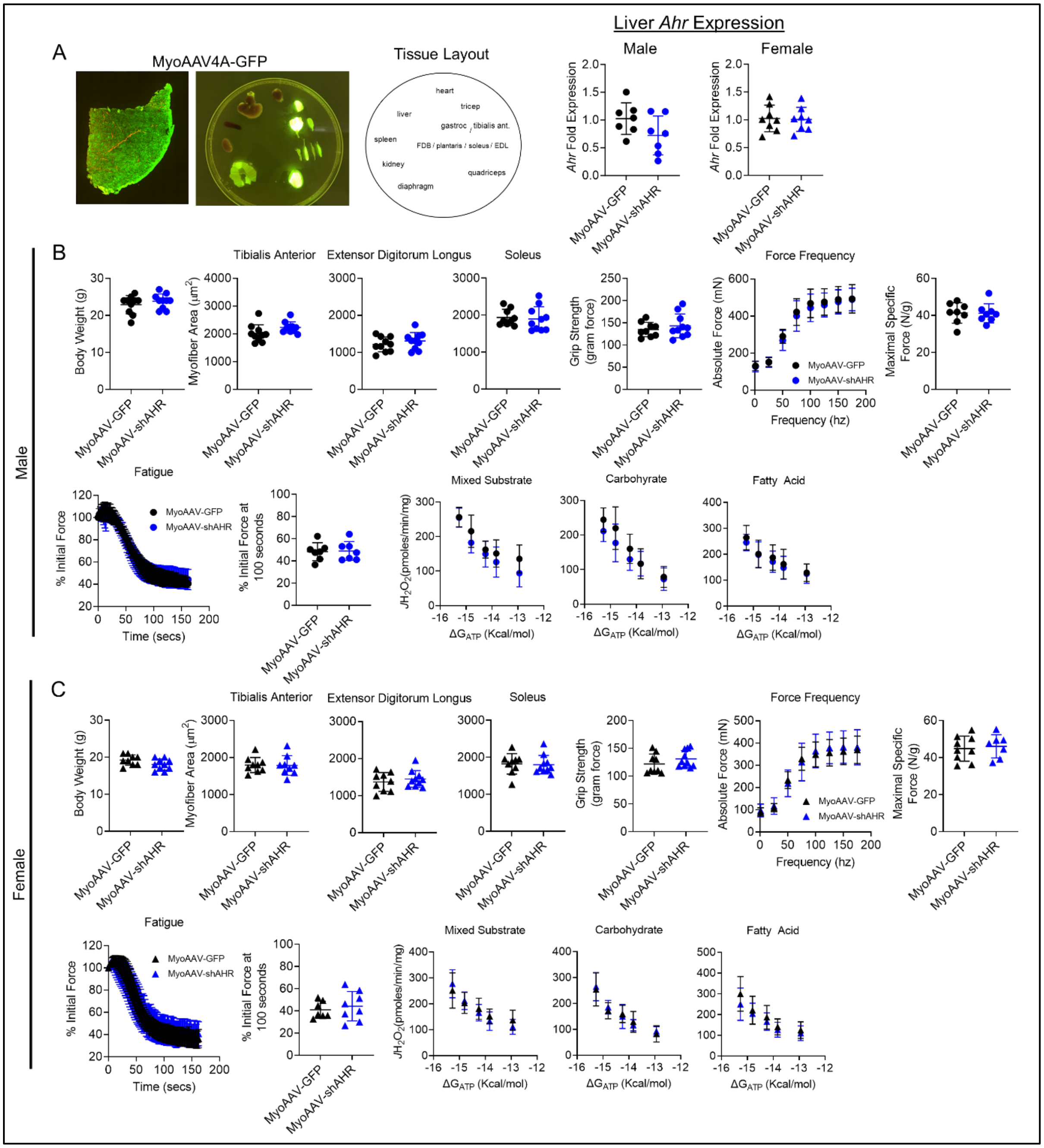

Supplemental Figure 7. (A) Validation of muscle specific expression using systemic delivery of MyoAAV4a-GFP and *Ahr* gene expression in mouse livers. (B) Body weights, muscle wet weights (tibialis anterior, extensor digitorum longus, and soleus), *in vivo* dual forelimb grip strength, *in* situ peroneal nerve stimulated force frequency (absolute force) and maximal specific force production of the extensor digitorum longus, quantification of *in situ* extensor digitorum longus fatigue, and the relationship between JH_2O_2 and ΔG_{ATP} in isolated mitochondria from the gastrocnemius energized with multiple substrate combinations in CKD males with either MyoAAV-GFP or MyoAAV-shAHR (n=7-10/group). (C) Identical measurements in female mice (n=7-10/group). Statistical analyses performed using Student's *t*-test. Error bars represent standard deviation.

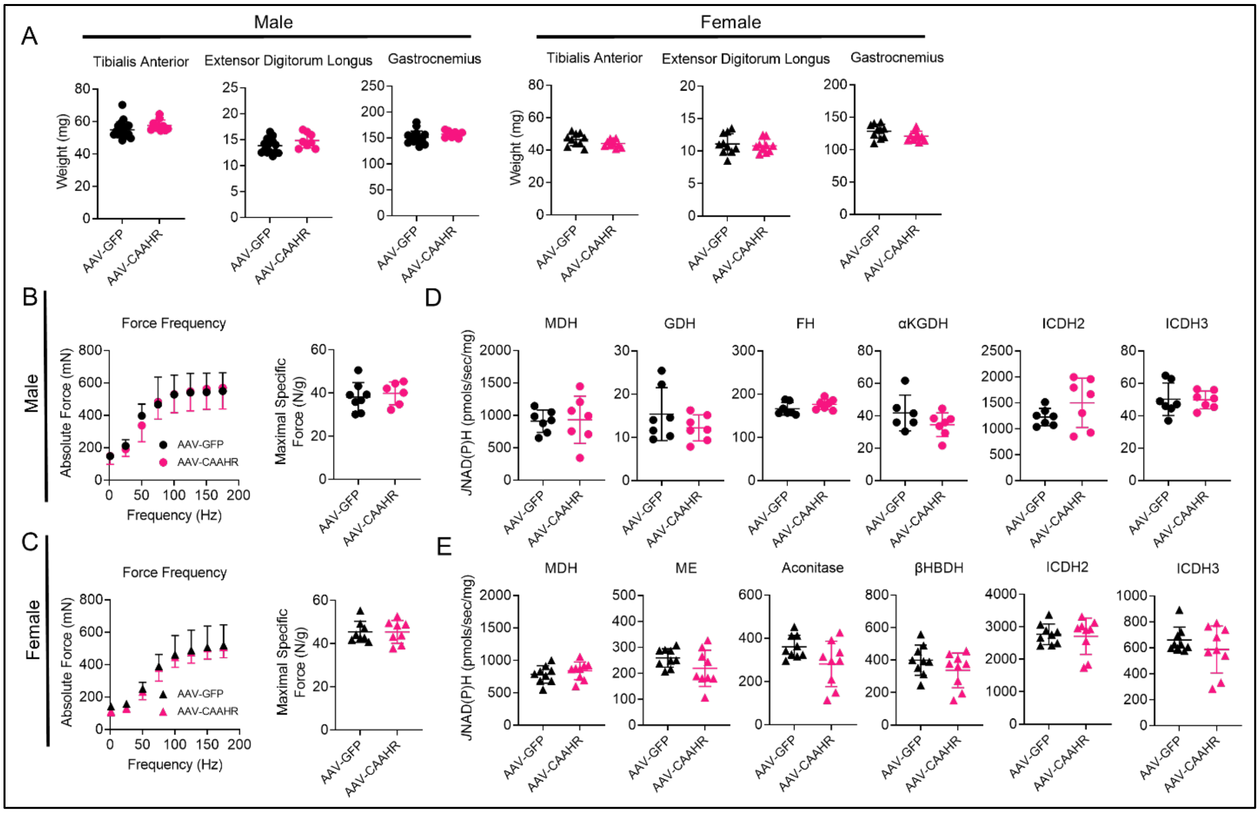

Supplemental Figure 8. (A) Muscle wet weights in male and female AAV-CAAHR mice compared to AAV-GFP controls (n=7-19/group). (B) *In situ* extensor digitorum longus force frequency (absolute force) and maximal specific force in males (n=6-8/group) and (C) females comparing AAV-GFP controls with AAV-CAAHR mice (n=8/group). (D) Quantification of mitochondrial matrix dehydrogenase activity in isolated mitochondrial from males (n=6-7/group) and (E) females (n=7-9/group) with ectopic expression of the AAV-CAAHR or AAV-GFP. Statistical analyses performed using Student's *t*-test and two-way ANOVA with Dunnett's post hoc testing for multiple comparisons when significant interactions were detected. Error bars represent the standard deviation. ***P*<0.01, *****P*<0.0001.

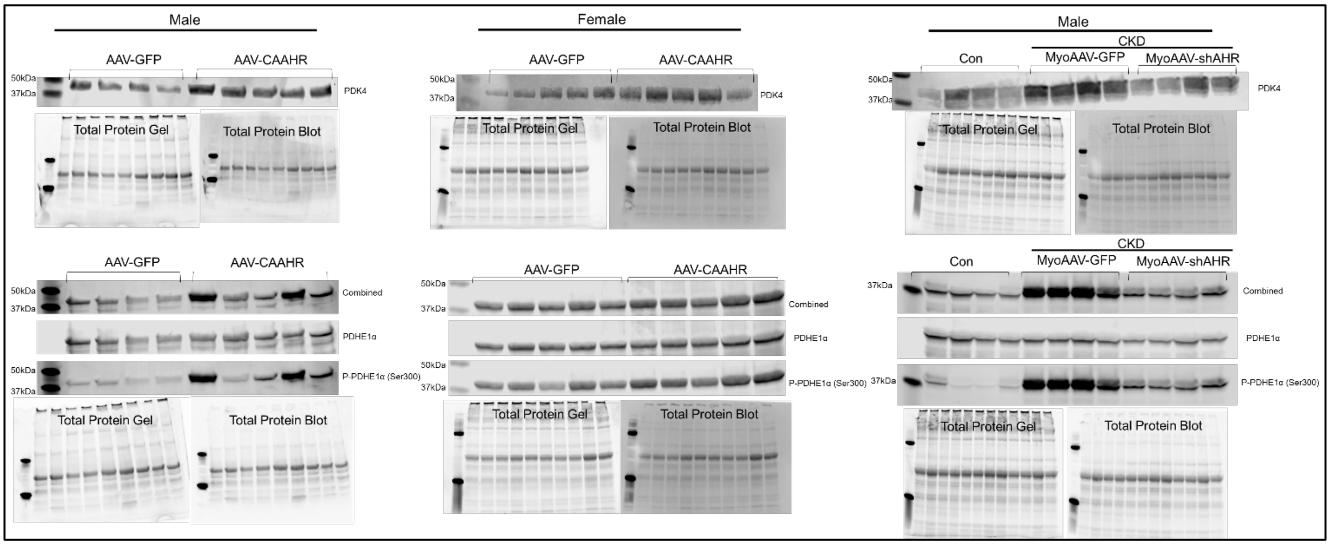

Supplemental Figure 9. Uncropped blots and gels related to data in Figure 6.

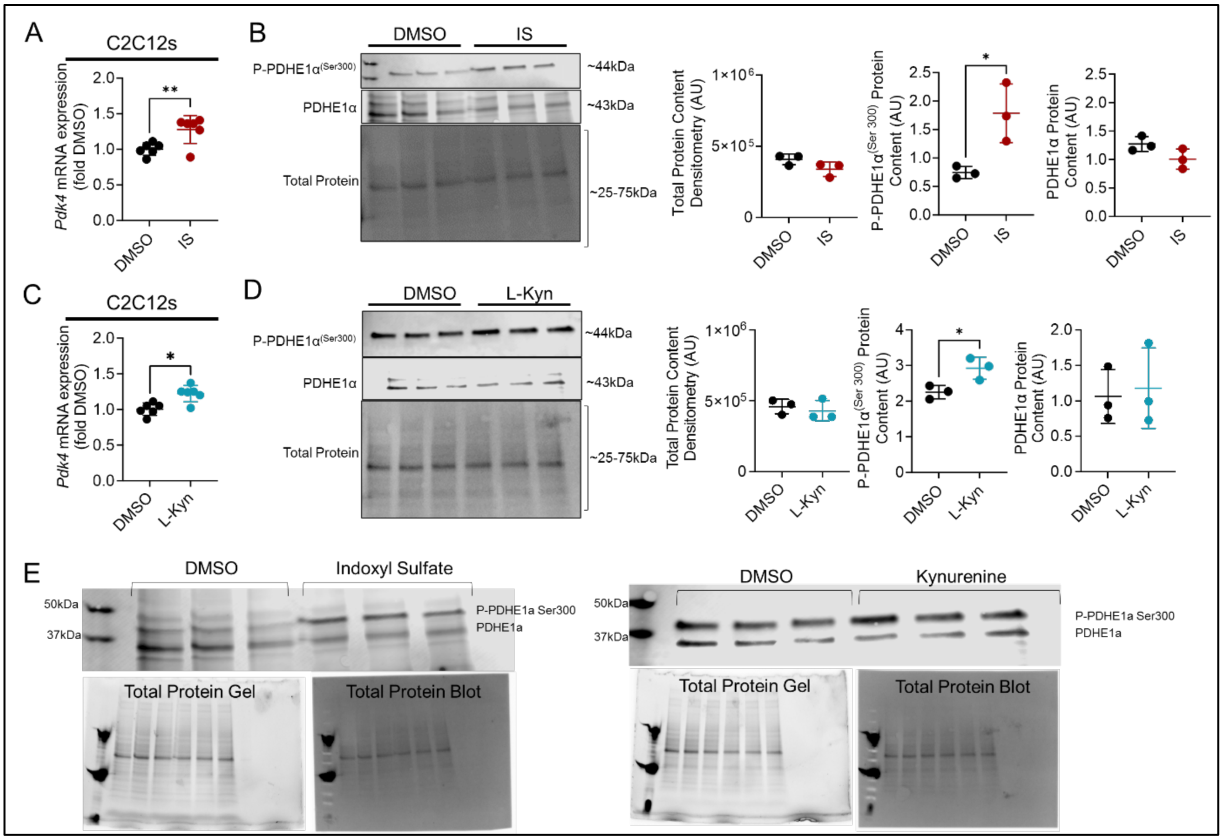

Supplemental Figure 10. AHR activation increased *Pdk4* expression and PDHE1α phosphorylation. (A) qPCR of *Pdk4* mRNA expression in C2C12 muscle cells treated with DMSO or indoxyl sulfate (IS, 100μM) (n=6/group). (B) Western blotting of phosphorylated and total PDHE1α protein expression in C2C12 muscle cells treated with DMSO or indoxyl sulfate (IS, 100μM) (n=3/group). (C) qPCR of *Pdk4* mRNA expression in C2C12 muscle cells treated with DMSO or L-Kynurenine (L-Kyn, 100μM) (n=6/group). (D) Western blotting of phosphorylated and total PDHE1α protein expression in C2C12 muscle cells treated with DMSO or L-Kynurenine (L-Kyn, 100μM) (n=3/group). (E) Uncropped blots and gels related to data in (B) and (D). Data analyzed using two-tailed Student's *t*-test. Error bars represent standard deviation. *$P<0.05$, **$P<0.01$.
